# Objective understanding of the Nutri-score front-of-pack label by European consumers and its effect on food choices: an online experimental study

**DOI:** 10.1186/s12966-020-01053-z

**Published:** 2020-11-19

**Authors:** Manon Egnell, Zenobia Talati, Pilar Galan, Valentina A. Andreeva, Stefanie Vandevijvere, Marion Gombaud, Louise Dréano-Trécant, Serge Hercberg, Simone Pettigrew, Chantal Julia

**Affiliations:** 1 Sorbonne Paris Nord University, Inserm, Inrae, Cnam, Nutritional Epidemiology Research Team (EREN), Epidemiology and Statistics Research Centre -University of Paris (CRESS), Bobigny, 93000 France; 2 School of Psychology, Curtin University, Kent St, Bentley, WA 6102 Australia; 3 Scientific Institute of Public Health (Sciensano), J.Wytsmanstraat 14, 1050 Brussels, Belgium; 4 Department of Public Health, Hôpitaux Universitaires Paris Seine-Saint-Denis (AP-HP), Bobigny, 93000 France; 5 The George Institute for Global Health, Newtown NSW, Sydney, 2042 Australia

**Keywords:** Front-of-pack nutrition label, Objective understanding, Food choices, European consumers

## Abstract

**Background:**

The effectiveness of Front-of-Pack nutrition Labels (FoPLs) may be influenced by national context. In light of the ongoing efforts to harmonize FoPLs across Europe, this study aimed to compare the effectiveness of five FoPLs (Health Star Rating system, Multiple Traffic Lights, Nutri-Score, Reference Intakes, Warning symbols) on consumer understanding and food choice in 12 European countries.

**Methods:**

In 2018–2019, for three food categories, approximately 1000 participants per country were asked to select which food they would prefer to purchase between three products with distinct nutritional quality profiles, and then to rank the products by nutritional quality. Participants (*N* = 12,391 in total) completed these tasks first with no FoPL and then, after randomization to one of the five FoPLs, with a FoPL on the food packages. Associations between FoPLs and change in (i) nutritional quality of food choices and (ii) ability to correctly rank the products by nutritional quality were assessed with logistic regression models adjusted for sociodemographic and lifestyle characteristics of participants, conducted overall and by country.

**Findings:**

Compared with the Reference Intakes, the Nutri-Score (OR = 3.23[2.75–3.81]; *p* < 0.0001), followed by the Multiple Traffic Lights (OR = 1.68[1.42–1.98]; *p* < 0.0001), was the most effective FoPL in helping consumers identify the foods’ nutritional quality, overall and in each of the 12 countries. Differences between FoPLs regarding food choice modifications were smaller, but the effect of the Nutri-Score seemed slightly higher in eliciting healthier food choices overall compared with the Reference Intakes, followed by the Warning symbols, the Multiple Traffic Lights and the Health Star Rating system.

**Interpretation:**

In the context of FoPL harmonization in Europe, these findings from an online experiment provide insights into the Nutri-Score’s effectiveness on European consumers.

**Supplementary Information:**

The online version contains supplementary material available at 10.1186/s12966-020-01053-z.

## Introduction

Front-of-Pack nutrition Labels (FoPL) have been identified as potential tools to improve the dietary habits of populations and thus help prevent obesity and non-communicable diseases [1]. Along with helping consumers interpret the nutritional quality of food products by providing simplified nutritional information [2, 3], FoPLs can improve the nutritional quality of the food supply through the reformulation and innovation of food products by manufacturers [4].

In recent decades, multiple voluntary FoPL schemes have been implemented worldwide, including in several European countries, with some endorsed by governments while others have been developed as initiatives by food manufacturers or non-governmental organisations [5]. Thus, given the European regulation on nutritional labelling, the presence of multinationals and the free movements of goods across borders in Europe [6], multiple FoPL formats can currently be found on the European market, including the Green Keyhole in Nordic countries (since the 1990s), the Multiple Traffic Lights in the United Kingdom (UK) (since 2013), the Nutri-Score in France, Belgium, Spain, Germany, the Netherlands, Luxembourg and Switzerland (since 2017–2019), the warning symbols on salt content in Finland or the Reference Intakes developed by manufacturers (since 2006). All of these labels have been implemented in a voluntary manner as mandatory implementation of FoPL is currently not possible in Europe. A harmonization of FoPLs in Europe is thus currently being discussed in order to prevent confusion among consumers and simplify for manufacturers the distribution of goods across multiple countries [5]. To help governments and the European Commission make an informed decision when selecting an existing or new FoPL in Europe, it appears of major importance to investigate in different European countries the relative effectiveness of FoPLs, in particular because the Nutri-Score is being considered in a growing number of countries and is supported by consumers associations and a growing number of food retailers and food manufacturers [7].

FoPLs implemented worldwide and notably across Europe vary according to their graphical format, using a nutrient-specific (i.e. displaying information on specific nutrients, generally unfavourable) or summary (i.e. indicating the overall nutritional quality of foods) approach, using a colour-coded or monochrome format, and varying in the degree of interpretive aids they provide [8]. In 2007, a theoretical framework was published to summarize the different key steps of FoPL use, including visual perception, attitudes, understanding, and effect on food choices [9]. Most of the studies on FoPL evaluation have observed that the graphical format could influence the different dimensions of FoPL effectiveness. Research to date indicates that interpretive FoPLs proving guidance to consumers to interpret the nutritional quality of foods – through the use of symbols or colours for example, as is the case for the Multiple Traffic Lights, the Nutri-Score, the Warning symbols, or the Health Star Rating system – are well perceived by consumers, better understood and more effective in encouraging healthier choices than purely informative labels displaying only numerical information such as the Guideline Daily Amounts, or the Reference Intakes [10–12]. However, most of the studies focused on older formats (e.g. Multiple Traffic Lights, Guidelines Daily Amounts or Reference Intakes), and fewer studies have investigated the effectiveness of recent schemes (e.g. Health Star Rating system, Nutri-Score, the Warning symbols), or have explored the effectiveness of these FoPLs across different countries. The literature suggests the potential influence of sociocultural contexts on understanding and use of FoPLs [13–15], making international comparisons important when attempting to identify FoPLs that would be appropriate for cross-country application. The present study compares the performance of the five FoPLs in terms of objective understanding of nutritional quality and food choices among consumers in 12 European countries participating in the FOP-ICE (Front-Of-Pack International Comparative Experimental) study [16, 17].

## Materials and methods

### Participants

Using an international accredited web panel provider (PureProfile), between April and July 2018 during the first wave of the FOP-ICE study, approximately 1000 participants per country were recruited in 12 countries, including the six following European countries: Bulgaria, Denmark, France, Germany, Spain, and the UK (*N* = 6013 participants). Then, between March and July 2019, approximately 1000 participants per country were also recruited from six additional European countries: Belgium, Italy, the Netherlands, Poland, Portugal, and Switzerland (*N* = 6378 participants). Thus, a final sample of 12,391 European participants was reached. These countries were selected for different reasons: (1) countries corresponded to various regions of Europe (Northern, Eastern, Western and Southern Europe); (2) some countries were facing public debates on front-of-pack labelling during the study; and (3) some countries in which a FoPL was already implemented were selected to enable assessment of the effects of familiarity with a scheme (i.e. the United Kingdom with the Multiple Traffic Lights, France and to a lesser extent Belgium with the Nutri-Score). To ensure equal coverage of main population sub-groups, recruitment was performed using a quota sampling method regarding gender (50% of women), age (one-third in each category of 18–30 years, 31–50 years and over 50 years), and socioeconomic status (one-third across low, medium and high household income levels). For each country, income level categories were calculated using the median household income of the country and creating a bracket of +/− 33% around this median. This represented the ‘intermediate’ income band. Incomes below or above corresponded respectively to the low- and high-income levels. To assess eligibility, participants were asked to report their purchasing frequency for the three food categories tested (pizzas, cakes and breakfast cereals). Individuals who declared never purchasing any of these products were ineligible to participate. The study protocol was approved by the Institutional Review Board of the French Institute for Health and Medical Research (IRB Inserm n°17–404 bis) and the Curtin University Human Research Ethics Committee (HRE2017–0760), and the written consent of all participants was obtained at the beginning of the questionnaire. The protocol can be found at https://www.anzctr.org.au/ACTRN12618001221246.aspx.

### Stimuli and procedure

#### Stimuli

Three food categories (1) displaying high variability in nutritional quality of products within the category and (2) commonly consumed in the different countries included were chosen, corresponding to pizzas, cakes, and breakfast cereals. For each category, a set of three products with clearly distinct nutrient profiles (lower, intermediate and higher nutritional quality) was developed: three pizzas (vegetarian pizza – Quattro Stagioni; mixed pizza – Regina; cheese pizza – Quattro Fromaggi), three cakes (cheesecake; brownie; poundcake), and three breakfast cereals (cornflakes; chocolate cereals; chocolate filled cereals). The stimuli were identical in the 12 countries and across conditions to enable cross-cultural comparisons of FoPL effectiveness in standardized conditions. Mock packages were created to resemble real food products but with a fictional brand (“Stofer”). In the second part of the study, FoPLs were affixed to the front of packages and covered roughly the same surface area on all food products. A zoom function was available to allow participants to enlarge any area of the package including the FoPL. No other information or quality indicators (e.g. nutrition or health claims, price, organic label) appeared on the mock packages to limit the influence of other factors on participants’ perceptions and choices.

#### Procedure

**Fig. 1:**
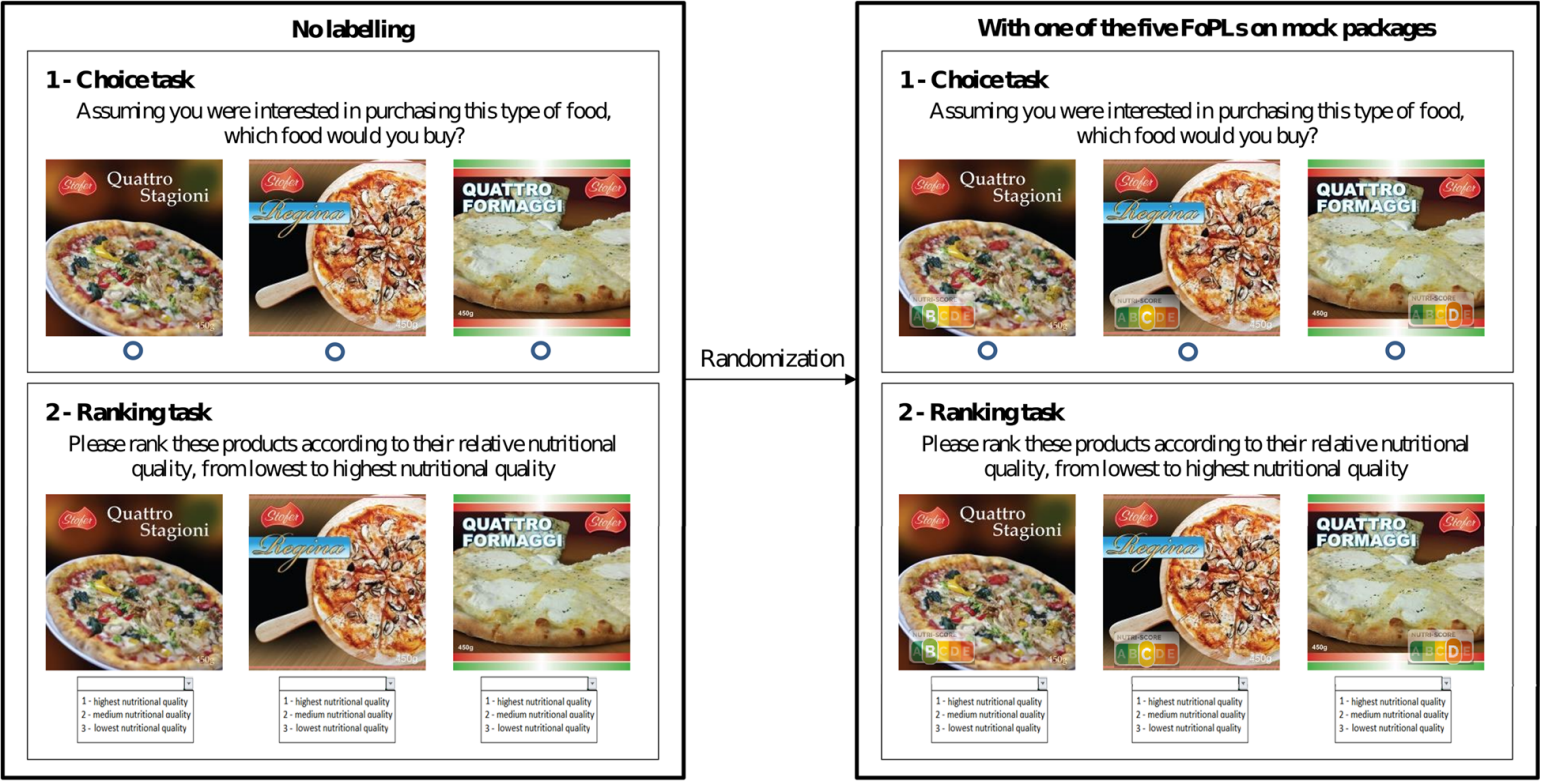
Procedures for the choice and ranking tasks for the pizza category

Participants were invited to respond to an online survey that was translated to the national language of each country. Participants were first invited to answer questions on gender, age, monthly household income, household composition, education level, purchasing frequency of the tested food categories, involvement in grocery shopping, self-estimated level of nutrition knowledge and self-assessed diet quality. Then, participants were invited to perform choice and ranking tasks. The food choice task was completed before assessing understanding to avoid priming effects. First, participants were asked to select the product within the set of three products without any FoPL they would be most likely to purchase. An “I wouldn’t buy any of these products” option was also available. Then, they were invited to rank the set of three products without any FoPL according to their nutritional quality by choosing for each product “1 – Highest nutritional quality”, “2 – Intermediate nutritional quality” or “3 – Lowest nutritional quality”. An “I don’t know” option was also available. Participants completed the choice and ranking tasks successively for the three food categories. They were then randomized to one of the five FoPLs and invited to repeat the choice and ranking tasks for the three categories. The expected ranking of the products within a set according to nutritional quality was similar whatever the FoPL affixed to the front of packages. The choice and ranking tasks for pizzas are provided as an illustrative example in Fig. 1 [18]. At the end of the questionnaire, participants were asked if they recalled having seen the label to which they were exposed. Any potential bias related to the presentation order of categories and products was controlled for by randomising the order in which the food categories and products within sets were presented.

#### Front-of-pack nutrition labels

The five FoPLs included in the present study are depicted in Fig. S1 [18]. Three nutrient-specific formats were tested, including (i) the Reference Intakes label, a purely numerical monochromatic scheme providing information on the amounts of energy and nutrients of concern (i.e. total fats, saturated fats, sugars and salt) per portion and in terms of contribution to the daily guideline intakes; (ii) the Multiple Traffic Lights, a colour-coded label displaying information on the content per portion of energy and the same nutrients of concern, with associated colours per nutrient (green for low, orange for medium and red for high amounts); (iii) the Warning symbols, black warning labels applied on products when the level of energy or a given nutrient of concern (saturated fats, sugars, sodium) exceeds what is considered a healthy amount. Two summary labels were also included in the study: (i) the Nutri-Score, a summary colour-coded scheme characterizing the overall nutritional quality of a product using a 5-colour scale going from green (associated with the letter A) to red (associated with the letter E) and (ii) the Health Star Rating system that uses a graded scale of stars combined with information on nutrient amounts.

### Outcomes and statistical analyses

#### Food choices

For each food category and labelling condition (no FoPL and with FoPL), choice was coded on a 3-point scale, from + 1 point for the product of the lowest nutritional quality, to + 3 points for the product of the highest nutritional quality. For each food category, a score was calculated as the difference between the FoPL and no FoPL conditions, ranging between − 2 (the highest possible deterioration in the nutritional quality of the food choice with the label compared to no label) to + 2 points (the highest possible improvement). For each participant, scores of the three food categories were summed to provide an overall discrete choice score ranging between − 6 and + 6 points. In descriptive analyses, the number of participants exhibiting deteriorated or improved choices was calculated for each category and each FoPL group. Then, in each country, the associations between the FoPLs and the change in the nutritional quality of choices were estimated using multivariable logistic regression models, adjusted for covariates, including sex, age, educational level, level of income, responsibility for grocery shopping, self-estimated diet quality and self-estimated nutrition knowledge level. These analyses included only participants who made a choice in the two labelling conditions. The FoPLs’ performances were compared in the model using the Reference Intakes as the reference category. For the overall sample, meta-analysis statistical method was used to assess the overall effect of FoPLs on food choices using a mixed ordinal logistic regression model with a random effect of the labels. Analyses were performed for the three food categories combined and by category. Sensitivity analyses were conducted with an additional adjustment on the response to the question “Did you see the label during the survey?”. Finally, other sensitivity analyses were performed by adjusting the overall models by food category on the corresponding purchasing frequency.

#### Objective understanding

Objective understanding of the FoPLs by participants was assessed by comparing the results to the ranking tasks in the two labelling conditions. The ranking was considered correct when the three products within a set were ranked in the expected order according to their nutritional quality. The number of correct responses in the two labelling conditions and the percentage of change between the two conditions were computed for each food category and FoPL group. Then, for each food category, − 1 point was given to the participant if the ranking was incorrect, 0 if the participant chose the “I don’t know” option and + 1 point if the participant correctly ranked the three products. Using the difference in points between the two labelling conditions, a score by food category ranging from − 2 to + 2 points was computed. Finally, an overall discrete understanding score was computed by summing the three food category scores, ranging from − 6 to + 6 points. The associations between FoPLs and the change in participants’ ability to correctly rank products according to nutritional quality were evaluated in each country using multivariable ordinal logistic regression, adjusted for the same covariates as the choice models, and the Reference Intakes was again used as the reference category. Similar to the choice analyses, a mixed ordinal regression model with a random effect of the label was used in the overall sample. Analyses were performed across and within food categories. Sensitivity analyses were conducted with an additional adjustment on the response to the question “Did you see the label during the survey?”. Sensitivity analyses were also performed, without discriminating participants having responded “I don’t know” to the ranking task from those having ranked at least one product out of the expected order, following previous methodology (1 point if the ranking was correct, 0 point if the participant responded “I don’t know” or ranked at least one product out of order) [16]. Similar to the choice analyses, other sensitivity analyses were performed by adjusting the overall models by food category on the corresponding purchasing frequency.

Interactions between FoPLs and nutrition-related individual characteristics (i.e., self-estimated diet quality and nutrition knowledge) on choice and understanding outcomes were tested. All analyses were performed with SAS statistical software. Statistical tests were two-sided and a *p*-value of ≤0.05 was considered statistically significant. The False Discovery Rate approach proposed by Benjamini and Hochberg (1995) was used to account for multiple testing.

## Results

### Description of the sample

The sociodemographic and lifestyle characteristics of the sample are described overall and by country in Table 1. Overall, 32.58% of participants had a primary or secondary school education level only, 71.50% reported being responsible for grocery shopping, 22.35% reported having a mostly or very unhealthy diet and 22.86% reported having little or no nutrition knowledge.

**Table 1:** Sociodemographic and nutrition-related lifestyle characteristics of the population sample, overall and by country, N(%)

	Belgium	Bulgaria	Denmark	France	Germany	Italy	Netherlands	Poland	Portugal	Spain	Switzerland	United Kingdom	Total
**N**	1007	1013	1000	1000	1000	1032	1032	1160	1059	1000	1088	1000	12,391
**Gender**													
Men	505 (50.15)	508 (50.15)	500 (50.00)	500 (50.00)	500 (50.00)	515 (49.90)	517 (50.10)	580 (50.00)	526 (49.67)	500 (50.00)	560 (51.47)	500 (50.00)	6211 (50.13)
Women	502 (49.85)	505 (49.85)	500 (50.00)	500 (50.00)	500 (50.00)	517 (50.10)	515 (49.90)	580 (50.00)	533 (50.33)	500 (50.00)	528 (48.53)	500 (50.00)	6180 (49.87)
**Age, years**													
18–30	336 (33.37)	359 (35.44)	328 (32.80)	333 (33.30)	340 (34.00)	347 (33.62)	345 (33.43)	390 (33.62)	364 (34.37)	339 (33.90)	342 (31.43)	332 (33.20)	4155 (33.53)
31–50	336 (33.37)	379 (37.41)	333 (33.30)	333 (33.30)	330 (33.00)	343 (33.24)	343 (33.24)	390 (33.62)	363 (34.28)	331 (33.10)	371 (34.10)	334 (33.40)	4186 (33.78)
> 50	335 (33.27)	275 (27.15)	339 (33.90)	334 (33.40)	330 (33.00)	342 (33.14)	344 (33.33)	380 (32.76)	332 (31.35)	330 (33.00)	375 (34.47)	334 (33.40)	4050 (32.69)
**Educational level**													
Primary education	55 (5.46)	6 (0.59)	94 (9.40)	17 (1.70)	97 (9.70)	16 (1.55)	13 (1.26)	20 (1.72)	11 (1.04)	21 (2.10)	68 (6.25)	7 (0.70)	425 (3.43)
Secondary education	328 (32.57)	142 (14.02)	172 (17.20)	183 (18.30)	382 (38.20)	240 (23.26)	314 (30.43)	474 (40.86)	354 (33.43)	316 (31.60)	326 (29.96)	381 (38.10)	3612 (29.15)
Trade certificate	117 (11.62)	252 (24.88)	391 (39.10)	266 (26.60)	241 (24.10)	259 (25.10)	277 (26.84)	122 (10.52)	139 (13.13)	166 (16.60)	371 (34.10)	144 (14.40)	2745 (22.15)
University, undergraduate degree	356 (35.35)	262 (25.86)	210 (21.00)	334 (33.40)	129 (12.90)	289 (28.00)	329 (31.88)	192 (16.55)	427 (40.32)	282 (28.20)	189 (17.37)	343 (34.30)	3342 (26.97)
University postgraduate degree	151 (15.00)	351 (34.65)	133 (13.30)	200 (20.00)	151 (15.10)	228 (22.09)	99 (9.59)	352 (30.34)	128 (12.09)	215 (21.50)	134 (12.32)	125 (12.50)	2267 (18.30)
**Level on household income**													
High	338 (33.57)	370 (36.53)	320 (32.00)	334 (33.40)	327 (32.70)	342 (33.14)	342 (33.14)	387 (33.36)	355 (33.52)	330 (33.00)	367 (33.73)	335 (33.50)	4147 (33.47)
Medium	340 (33.76)	359 (35.44)	340 (34.00)	333 (33.30)	333 (33.30)	343 (33.24)	343 (33.24)	397 (34.22)	355 (33.52)	330 (33.00)	371 (34.10)	335 (33.50)	4179 (33.73)
Low	329 (32.67)	284 (28.04)	340 (34.00)	333 (33.30)	340 (34.00)	347 (33.62)	347 (33.62)	376 (32.41)	349 (32.96)	340 (34.00)	350 (32.17)	330 (33.00)	4065 (32.81)
**Responsible for grocery shopping**													
Yes	738 (73.29)	599 (59.13)	690 (69.00)	863 (86.30)	769 (76.90)	765 (74.13)	746 (72.29)	834 (71.9)	640 (60.43)	747 (74.70)	718 (65.99)	750 (75.0)	8859 (71.5)
No	73 (7.25)	64 (6.32)	55 (5.50)	21 (2.10)	31 (3.10)	50 (4.84)	55 (5.33)	35 (3.02)	75 (7.08)	35 (3.50)	86 (7.90)	35 (3.50)	615 (4.96)
Share job equally	196 (19.46)	350 (34.55)	255 (25.50)	116 (11.60)	200 (20.00)	217 (21.03)	231 (22.38)	291 (25.09)	344 (32.48)	218 (21.80)	284 (26.10)	215 (21.50)	2917 (23.54)
**Self-estimated diet quality**													
I eat a very unhealthy diet	17 (1.69)	48 (4.74)	12 (1.20)	20 (2.00)	34 (3.40)	1 (0.10)	8 (0.78)	3 (0.26)	5 (0.47)	11 (1.10)	20 (1.84)	11 (1.10)	190 (1.53)
I eat a mostly unhealthy diet	213 (21.15)	609 (60.12)	199 (19.90)	182 (18.20)	202 (20.20)	104 (10.08)	102 (9.88)	253 (21.81)	147 (13.88)	162 (16.20)	196 (18.01)	211 (21.10)	2580 (20.82)
I eat a mostly healthy diet	634 (62.96)	341 (33.66)	727 (72.70)	660 (66.00)	677 (67.70)	787 (76.26)	865 (83.82)	851 (73.36)	855 (80.74)	711 (71.10)	769 (70.68)	715 (71.50)	8592 (69.34)
I eat a very healthy diet	143 (14.20)	15 (1.48)	62 (6.20)	138 (13.80)	87 (8.70)	140 (13.57)	57 (5.52)	53 (4.57)	52 (4.91)	116 (11.60)	103 (9.47)	63 (6.30)	1029 (8.30)
**Nutrition knowledge**													
I do not know anything about nutrition	31 (3.08)	9 (0.89)	10 (1.00)	51 (5.10)	15 (1.50)	3 (0.29)	7 (0.68)	0 (0)	6 (0.57)	26 (2.60)	22 (2.02)	17 (1.70)	197 (1.59)
I am not very knowledgeable about nutrition	287 (28.50)	210 (20.73)	166 (16.60)	408 (40.80)	193 (19.30)	132 (12.79)	157 (15.21)	168 (14.48)	104 (9.82)	287 (28.70)	288 (26.47)	235 (23.50)	2635 (21.27)
I am somewhat knowledgeable about nutrition	519 (51.54)	627 (61.90)	638 (63.80)	380 (38.00)	617 (61.70)	746 (72.29)	744 (72.09)	853 (73.53)	675 (63.74)	609 (60.90)	579 (53.22)	664 (66.40)	7651 (61.75)
I am very knowledgeable about nutrition	170 (16.88)	167 (16.49)	186 (18.60)	161 (16.10)	175 (17.50)	151 (14.63)	124 (12.02)	139 (11.98)	274 (25.87)	78 (7.80)	199 (18.29)	84 (8.40)	1908 (15.40)
**Did you see the FOP label during the survey?**													
No	277 (27.51)	311 (30.70)	351 (35.10)	321 (32.10)	306 (30.60)	316 (30.62)	293 (28.39)	336 (28.97)	271 (25.59)	275 (27.50)	313 (28.77)	256 (25.60)	3626 (29.26)
Unsure	110 (10.92)	139 (13.72)	75 (7.50)	75 (7.50)	140 (14.00)	68 (6.59)	133 (12.89)	228 (19.66)	132 (12.46)	150 (15.00)	105 (9.65)	90 (9.00)	1445 (11.66)
Yes	620 (61.57)	563 (55.58)	574 (57.40)	604 (60.40)	554 (55.40)	648 (62.79)	606 (58.72)	596 (51.38)	656 (61.95)	575 (57.50)	670 (61.58)	654 (65.40)	7320 (59.08)
**Participants who recalled seeing the FoPL they were exposed to**													
*Health Star Rating system*	101 (50.00)	85 (42.08)	105 (52.50)	103 (51.50)	90 (45.00)	108 (52.43)	111 (53.62)	104 (44.83)	111 (52.36)	82 (41.00)	122 (55.96)	109 (54.5)	1231 (49.66)
*Multiple Traffic Lights*	150 (74.63)	120 (59.11)	125 (62.50)	138 (69.00)	128 (64.00)	149 (72.33)	135 (65.53)	159 (68.53)	152 (71.70)	140 (70.00)	145 (66.82)	160 (80.00)	1701 (68.67)
*Nutri-Score*	155 (77.11)	152 (75.25)	131 (65.50)	130 (65.00)	136 (68.00)	130 (62.80)	147 (71.36)	108 (46.55)	145 (68.40)	107 (53.5)	164 (75.23)	138 (69.00)	1643 (66.30)
*Reference Intakes label*	133 (65.84)	112 (55.17)	133 (66.50)	131 (65.50)	128 (64.00)	162 (78.64)	136 (66.02)	154 (66.38)	149 (70.62)	155 (77.5)	143 (65.90)	153 (76.50)	1689 (68.19)
*Warning symbols*	81 (40.30)	94 (46.31)	80 (40.00)	102 (51.00)	72 (36.00)	99 (47.83)	77 (37.20)	71 (30.60)	99 (46.70)	91 (45.5)	96 (44.04)	94 (47.00)	1056 (42.58)

Overall, 59.08% of participants recalled having seen the FoPL they were exposed to during the survey, with homogeneous results across countries but heterogeneous results depending on the label. The Warning symbols (4.2.58%) and the health star rating system (49.66%) had the lowest proportions of participants recalling having seen the labels throughout the survey. The average duration of the online survey was 13 min.

### Food choices

Overall, across the five FoPL groups the percentage of participants improving the nutritional quality of their choices in the labelled condition compared to no label was higher than the percentage of participants who selected a product with lower nutritional quality with the FoPL compared to no label (Fig. S2). While the deterioration results were similar across FoPLs (between 3.5 and 4.9% of choices), the Nutri-Score appeared to lead to the highest percentage of participants improving their choices (between 7.7 and 11.2% across food categories), followed by the Multiple Traffic Lights (between 6.3 and 10.4%). The relative performance of the other FoPLs varied across food categories.

**Fig. 2:**
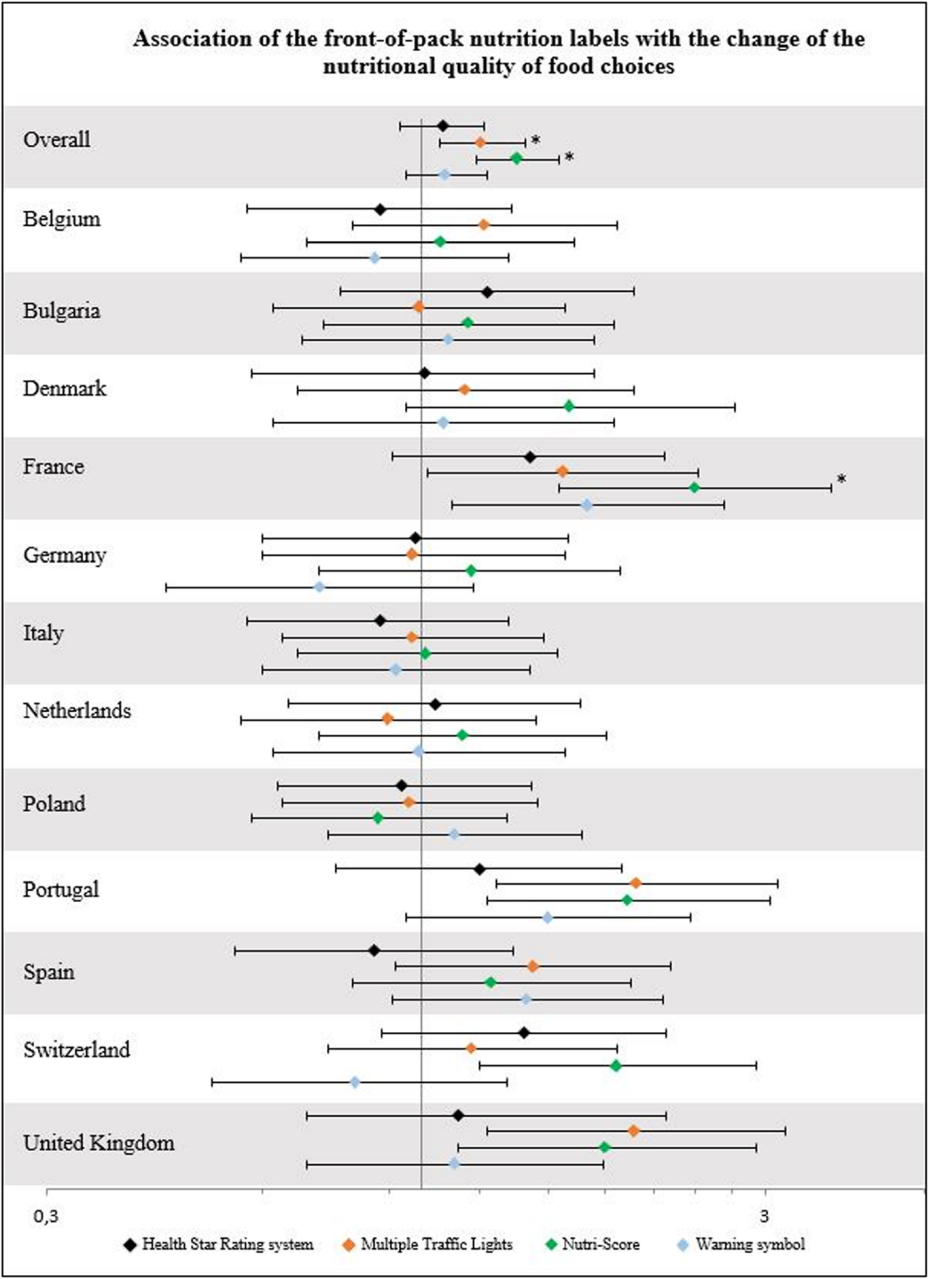
Changes in the nutritional quality of food choices between the FoPL and no-FoPL labelling conditions, compared to the Reference Intakes label. ^*^ Significant results (*p*-value≤0.05) after False Discovery Rate correction for multiple testing modifying the *p*-value. The reference of the multivariate ordinal logistic regression for the categorical variable ‘FoPL’ was the Reference Intakes label. The multivariate model was adjusted on sex, age, educational level, level of income, responsibility for grocery shopping, self-estimated diet quality, and self-estimated nutrition knowledge level. FoPL: Front-of-Pack nutrition Label

In the overall sample and the three food categories combined, compared to the Reference Intakes, the Nutri-Score was associated with the highest improvement in the nutritional quality of food choices (Odds Ratio OR = 1.36 [95% confidence Interval 1.19–1.55], *p*-value = 0.0001), followed by the Multiple Traffic Lights (OR = 1.21 [1.06–1.39], *p*-value = 0.02) (Fig. 2). The Warning symbols and the Health Star Rating system did not demonstrate any significant effect compared to the Reference Intakes. When analyses were performed by country, a significant positive association was only found for the Nutri-Score in France (OR = 2.40 [1.55–3.71], *p*-value = 0.02) after correction for multiple testing. When analyses were performed by food category, similar trends were observed overall for the Nutri-Score (Table S1 and S2). Within food categories, the Nutri-Score was the only FoPL to show a significant positive effect among pizzas and breakfast cereals on the food choices of the overall sample. In sensitivity analyses with an additional adjustment for the response to the question “Did you see the label during the survey?”, associations were strengthened with a significant effect of the Nutri-Score, followed by the Warning symbol, the Multiple Traffic Lights, and the Health Star Rating system, compared to the Reference Intakes (Table S3). Similar results were observed when models were adjusted on the food category purchasing frequency (Table S4). No interaction was found between the FoPL outcomes and nutrition-related individual characteristics.

### Objective understanding

**Fig. 3:**
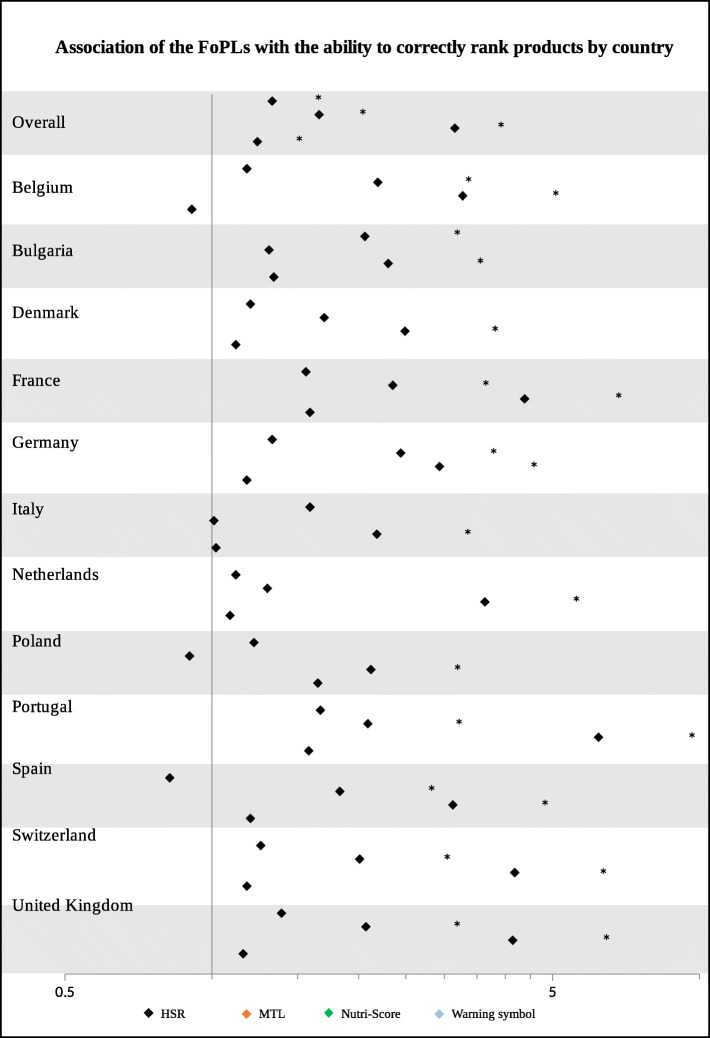
Changes in ability to correctly rank products between the FoPL and no-FoPL labelling conditions, compared to the Reference Intakes label, ^*^ Significant results (*p*-value≤0.05) after False Discovery Rate correction for multiple testing modifying the *p*-value. The reference of the multivariate ordinal logistic regression for the categorical variable ‘FoPL’ was the Reference Intakes label. The multivariate model was adjusted on sex, age, educational level, level of income, responsibility for grocery shopping, self-estimated diet quality, and self-estimated nutrition knowledge level. FoPL: Front-of-Pack nutrition Label

All FoPLs improved the number of correct answers compared to no label; however, large disparities were observed between FoPLs (Fig. S3). The Nutri-Score demonstrated the highest percentage of improvement in the number of correct answers, followed by the Multiple Traffic Lights. In the overall sample and the three food categories combined, all FoPLs were significantly more efficient than the Reference Intakes in improving participants’ ranking ability, with heterogeneous results depending on the label format (Fig. 3). Indeed, compared to the Reference Intakes, the Nutri-Score demonstrated the best performance (OR = 3.15 [2.68–3.71], *p*-value< 0.0001), followed by the Multiple Traffic Lights (OR = 1.66 [1.41–1.95], *p*-value< 0.0001), the Health Star Rating system (OR = 1.33 [1.14–1.57]; *p*-value = 0.002), and then the Warning symbols (OR = 1.24 [1.06–1.45], *p*-value = 0.02). When analyses were performed by country, the Nutri-Score remained the FoPL demonstrating the best performance in all 12 countries (between OR = 2.12 [1.49–3.02], *p*-value = 0.0006 for Poland and OR = 6.21 [4.27–9.04], *p*-value< 0.0001 for Portugal), while the relative performance of other FoPLs varied across countries (Table S5 and S6). The significant overall effect of FoPLs appears to be mainly driven by a positive effect in the cakes category, even though the Nutri-Score showed a positive effect for all three categories (Table S5 and S6). In sensitivity analyses adjusted for the response to the question “Did you see the label during the survey?” or without a distinction between incorrect ranking and no response, similar results were observed with consistency of the relative performance of FoPLs (Tables S7 and S8). When models were adjusted on the food category purchasing frequency, similar results were observed (Table S9). Similar to the choice analyses, no interaction was found between the FoPL outcomes and nutrition-related individual characteristics.

## Discussion

In the present study, compared to the Reference Intakes, the Nutri-Score demonstrated the greatest ability to help consumers rank the nutritional quality of foods, followed by the Multiple Traffic Lights, the Health Star Rating system and the Warning symbols. While similar trends were observed for the Nutri-Score in all 12 countries, the performance of the other FoPLs varied by country. Regarding the effect on food choices, differences between FoPLs were much smaller; nevertheless, for some of the countries (but significant results for France only) the Nutri-Score appeared to be also the most effective in improving the nutritional quality of food choices compared to the Reference Intakes. These findings are in line with the results observed in the other countries included in the first wave of the FOP-ICE study, especially in regard to objective understanding [16, 17].

Consistent with our findings, it has been found in the literature that interpretive FoPLs that provide guidance via their graphical format were more easily understood by consumers compared to purely informative labels (e.g. the Reference Intakes) [10]. In line with other studies [12, 16, 19], the Nutri-Score, followed by the Multiple Traffic Lights, was the FoPL associated with the largest improvement in participants’ ability to correctly rank the nutritional quality of foods, both overall and in the different individual countries included in the present study. The strong objective understanding results for the Nutri-Score followed by the Multiple Traffic Lights may be partly explained by the colour-coding used within these schemes, both of which use the green-red polychromatic scale. Colour-coding is likely to increase label salience, reducing the time needed by consumers to detect the information [20]. Second, colour-coding could help the interpretation of the information conveyed by the label, a later stage of information processing. In many countries, green reflects a “go” signal while red represents a “stop” signal, associations that are used in front-of-pack nutritional labelling and universally understood by consumers [21]. This could partly explain the higher performance of the Nutri-Score and the Multiple Traffic Lights compared to monochromatic formats (Health Star Rating system and the Warning symbols). Additional works could be conducted testing the effectiveness of different variants of a FoPL (colour-coded vs. monochrome) in order to better assess the insight of colour-use on consumers’ response [13, 22–24]. In addition, the superior performance of the Nutri-Score compared to the Multiple Traffic Lights may be related to the use of a summary indicator rather than a nutrient-specific format. Indeed, it has been suggested in the literature that summary schemes might be associated with a lower cognitive workload, while formats providing numerical information only require more time to process information and could lead to potential confusion about nutritional terms [12, 25].

Multiple studies have investigated the effect of FoPLs on food choices and purchases, with results suggesting that interpretive systems, such as the Nutri-Score [12], Multiple Traffic Lights [12, 26–28], the Health Star Rating [12, 27], and warning labels [29–33], may be particularly effective in encouraging healthier food choices. In our study, FoPLs seemed to improve the nutritional quality of food choices compared to no label, but with small differences between FoPLs. Nevertheless, the Nutri-Score showed the best results overall compared to the Reference Intakes. These findings might be considered with respect to the framework of Grunert et al., stating that the understanding of a FoPL can affect food choices [9]. Therefore, the higher performance of the Nutri-Score in helping the participants assess the relative nutritional quality of foods could partly explain its slightly larger impact on choices. However, it is important to note that the magnitude of the differences between FoPLs was much smaller regarding food choices than objective understanding. The methodology used might partly account for these results, given that the choice task pertained to a limited set of food products/categories. Indeed, it has been suggested that results of choice tasks might be influenced by the categories of products as well as the extent of product selection within the choice set [13].

In our experimental study, similar patterns of FoPL effects on food choice and understanding by consumers were observed in the different countries included, consistent with previously published results [16, 17, 34]. In the 12 countries, the Nutri-Score was the FoPL associated with the highest objective understanding by consumers. This could be related to its two graphical features: the summary indicator, and the use of colour-coding, which is universally understood by individuals. Although similar trends in the relative performance of FoPLs were observed across countries, the effect amplitudes were slightly different. Most of the countries in the present study that demonstrated a particularly strong association between the Nutri-Score and objective understanding compared to the Reference Intakes have recently been discussing the potential implementation of a national FoPL, with the Nutri-Score being considered as a viable option (i.e. France, Switzerland, the Netherlands, Portugal, Spain). According to the literature, the role of the public debate about nutrition, the national context and history regarding nutritional labelling and especially front-of-pack labelling, as well as potential media debate, might influence consumers’ responses to FoPLs in any given country [13–15]. The debates related to FoPL implementation might have been reflected in the choice analyses as well, but to a lesser extent. However, the potential influence of the media and public debates on FoPL effectiveness could not be measured in the present experiment. No clear pattern was observed for the other FoPLs that were tested in the present study. Finally, the Nutri-Score with its key graphical features seemed to outweigh any potential familiarity effects, given that it also showed stronger performance in the UK compared to the nutrient-specific Multiple Traffic Lights, which was implemented in that country in 2005.

This study provides more insights on the effectiveness of five FoPLs currently implemented worldwide, including the main types of label graphical format (i.e. monochromatic versus colour-coded, summary versus nutrient-specific) and using a randomization design, in multiple European countries. The recruitment strategy using quota sampling allowed us to balance the sample in each country and to reach individuals of various sociodemographic profiles, including low-income individuals who are difficult to access in research and for which the effectiveness of FoPLs could vary, rather than obtain representative samples in each country. In addition, this approach provided similar samples across countries thus enabling cross-cultural comparisons of FoPL effectiveness. However, caution is therefore required regarding the extrapolation of the present findings. Finally, a potential learning effect during the survey was limited by randomizing the order of (i) the food categories and (ii) the products within the sets. While learning effects could not be completely eradicated, any potential bias would have influenced the five FoPLs equally and thus would not have modified the relative performance of the schemes. However, some limitations should be acknowledged. First, despite the inclusion of various sub-groups of populations, recruitment via quota sampling resulted in samples that may not be representative of the populations in the various countries. In addition, participation in the survey was voluntary and the percentages of individuals reporting having a healthy diet and being knowledgeable about nutrition were high. These limitations indicate a need for caution when extrapolating the results. Second, participants were blind to the study objectives and no information was provided on the meaning of the FoPLs, which may have impacted the interpretation of the provided information. Nevertheless, our objective was to compare the FoPLs, and these potential biases affected all FoPLs equally. Third, preferences of participants for some food products may have influenced their food choices or their ability to identify and rank the nutritional quality of products, but were not assessed in the present study. However, this potential bias would be similar whatever the FoPL and would not have affected the relative performances of the various schemes. This aspect was confirmed by the similar trends observed in sensitivity analyses adjusted for the purchasing frequency of food categories, reflecting participants’ preferences. Finally, the study was conducted in experimental conditions, which differ from real-life settings where additional factors such as price may influence consumers’ food choices, and inferences about missing information could have been made by participants. Therefore, the findings of the study on FoPLs’ effectiveness have to be taken with caution and only hold notably for equally priced foods with different nutrient profiles. Even if virtual purchasing behaviours have been suggested to be good predictors of real behaviours [35], intentions can differ from real food behaviours [36, 37], and some real-life studies have suggested that FoPLs could be effective under specific condition [38, 39]. Investigating the effects of FoPLs on actual food purchases and real-life environments would therefore provide more definitive conclusions as to the various formats’ real impact to complement experimental findings. Nevertheless, the experimental online design allowed the study to be conducted in standardized conditions in all countries and for cross-cultural comparisons to be performed, while accommodating logistical and financial constraints.

Among the five FoPLs tested in the present experiment, the Nutri-Score, closely followed by the Multiple Traffic Lights and the Warning symbols, emerged as the most effective FoPL in terms of helping European consumers assess the nutritional quality of products and potentially encouraging them towards healthier food choices. This study provides insights on the effectiveness of five FoPLs already implemented worldwide in multiple European countries and the findings are particularly important for the current debate about harmonization of front-of-pack nutritional labelling in Europe, with the announcement of the European Commission to select a single FoPL in Europe in 2022 as part of the ‘Farm to Fork’ strategy from the Green Deal. While the Nutri-Score is implemented or considered by a growing number of European countries, some alternatives are proposed by opponents, such as the NutrInform Battery scheme – a variant of the Reference Intakes label – supported by the Italian government. International scientific studies are thus needed to confirm the effectiveness of summary colour-coded FoPLs, such as the Nutri-Score, in multiple European countries, especially from Northern and Eastern Europe, and assess other schemes such as the Italian NutrInform Battery, whose original format – the Reference Intakes – has shown no effect on consumer behaviours in most studies where it has been evaluated. Further research is also needed on the effectiveness of these FoPLs on food purchases of European consumers, especially in real-life conditions.

## Supplementary Information

**Additional file 1:** **Fig. S1.** Front-of-pack nutrition labels tested in the present study. **Fig. S2.** Percentage of participants having deteriorated or improved their food choices between the two labelling conditions (without and with FoPL), for the overall sample. **Fig. S3.** Percentage of correct answers in the two labelling conditions (without and with FoPL), for the overall sample. **Table S1.** Associationsa between FoPLs and the change in nutritional quality of food choices, across and within the three food categories. **Table S2.** Effect sizes of the associations between FoPLs and the change in nutritional quality of food choices, across and within the three food categories. **Table S3.** Associationsa between FoPLs and the change in nutritional quality of food choices, across and within the three food categories, adjusted on the response to “Did you see the label during the survey?”. **Table S4.** Associationsa between FoPLs and the change in nutritional quality of food choices, adjusted on food category purchasing frequency. **Table S5.** Associationsa between FoPLs and the change in participants’ ability to correctly rank the nutritional quality of foods, across and within the three food categories. **Table S6.** Effect sizes of the associations between FoPLs and the change in participants’ ability to correctly rank the nutritional quality of foods, across and within the three food categories. **Table S7.** Associationsa between FoPLs and the change in participants’ ability to correctly rank the nutritional quality of foods, across and within the three food categories, adjusted on the response to “Did you see the label during the survey?”. **Table S8.** Associationsa between FoPLs and the change in participants’ ability to correctly rank the nutritional quality of foods, across and within the three food categories, with no distinction between non-response and incorrect ranking. **Table S9.** Associationsa between FoPLs and the change in participants’ ability to correctly rank the nutritional quality of foods, adjusted on food category purchasing frequency.

## Data Availability

All data supporting the findings of this study are included in the present article or the supplemental material.
