# Correction to: Objective understanding of the Nutri-score front-of-pack label by European consumers and its effect on food choices: an online experimental study

**DOI:** 10.1186/s12966-020-01069-5

**Published:** 2020-12-16

**Authors:** Manon Egnell, Zenobia Talati, Pilar Galan, Valentina A. Andreeva, Stefanie Vandevijvere, Marion Gombaud, Louise Dréano-Trécant, Serge Hercberg, Simone Pettigrew, Chantal Julia

**Affiliations:** 1 Inserm, Inrae, Cnam, Nutritional Epidemiology Research Team (EREN), Epidemiology and Statistics Research Centre -University of Paris (CRESS), Sorbonne Paris Nord University, 93000 Bobigny, France; 2 School of Psychology, Curtin University, Kent St, Bentley, WA 6102 Australia; 3 Scientific Institute of Public Health (Sciensano), J.Wytsmanstraat 14, 1050 Brussels, Belgium; 4 Department of Public Health, Hôpitaux Universitaires Paris Seine-Saint-Denis (AP-HP), 93000 Bobigny, France; 5 The George Institute for Global Health, Newtown NSW, Sydney, 2042 Australia

**Correction to: Int J Behav Nutr Phys Act 17, 146 (2020)**

**https://doi.org/10.1186/s12966-020-01053-z**

Following the publication of the original article [1], the authors identified an error in Fig. 3. The correct figure is given below.

**Fig. 3:**
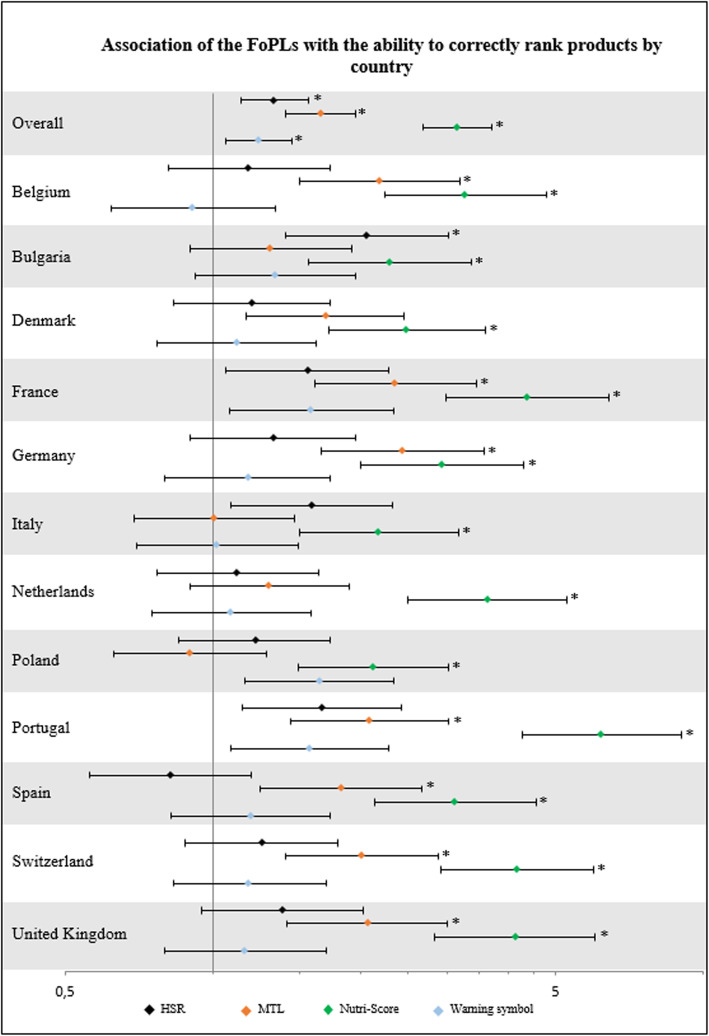
Changes in ability to correctly rank products between the FoPL and no-FoPL labelling conditions, compared to the Reference Intakes label, ^*^ Significant results (*p*-value≤0.05) after False Discovery Rate correction for multiple testing modifying the *p*-value. The reference of the multivariate ordinal logistic regression for the categorical variable ‘FoPL’ was the Reference Intakes label. The multivariate model was adjusted on sex, age, educational level, level of income, responsibility for grocery shopping, self-estimated diet quality, and self-estimated nutrition knowledge level. FoPL: Front-of-Pack nutrition Label

The original article [1] has been corrected.
