# Supplementary material for: Objective understanding of the Nutri-score front-of-pack label by European consumers and its effect on food choices: an online experimental study
Source: Int J Behav Nutr Phys Act. 2020 Nov 19;17:146. doi: 10.1186/s12966-020-01053-z (PMC7678195; doi:10.1186/s12966-020-01053-z)
Supplement: Supplementary file 1 — Additional file 1: Fig. S1. Front-of-pack nutrition labels tested in the present study. Fig. S2. Percentage of participants having deteriorated or improved their food choices between the two labelling conditions (without and with FoPL), for the overall sample. Fig. S3. Percentage of correct answers in the two labelling conditions (without and with FoPL), for the overall sample. Table S1. Associationsa between FoPLs and the change in nutritional quality of food choices, across and within the three food categories. Table S2. Effect sizes of the associations between FoPLs and the change in nutritional quality of food choices, across and within the three food categories. Table S3. Associationsa between FoPLs and the change in nutritional quality of food choices, across and within the three food categories, adjusted on the response to “Did you see the label during the survey?”. Table S4. Associationsa between FoPLs and the change in nutritional quality of food choices, adjusted on food category purchasing frequency. Table S5. Associationsa between FoPLs and the change in participants’ ability to correctly rank the nutritional quality of foods, across and within the three food categories. Table S6. Effect sizes of the associations between FoPLs and the change in participants’ ability to correctly rank the nutritional quality of foods, across and within the three food categories. Table S7. Associationsa between FoPLs and the change in participants’ ability to correctly rank the nutritional quality of foods, across and within the three food categories, adjusted on the response to “Did you see the label during the survey?”. Table S8. Associationsa between FoPLs and the change in participants’ ability to correctly rank the nutritional quality of foods, across and within the three food categories, with no distinction between non-response and incorrect ranking. Table S9. Associationsa between FoPLs and the change in participants’ ability to correctly rank the nutritional qualit [file 12966_2020_1053_MOESM1_ESM.docx]

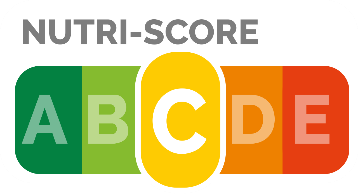

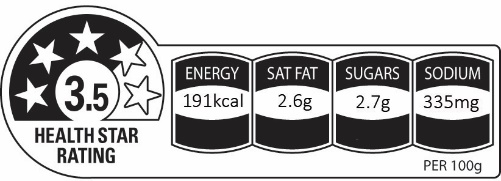

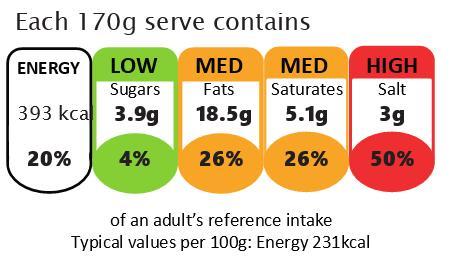

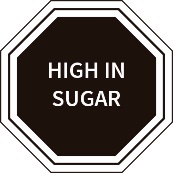

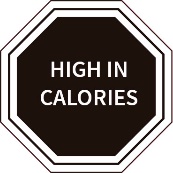

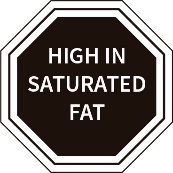

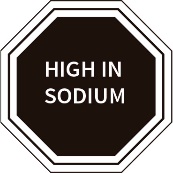

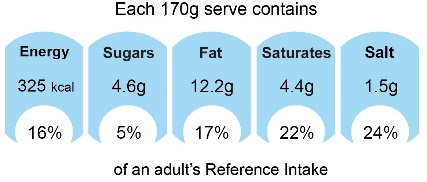


Multiple Traffic Lights

Reference Intakes

Warning symbol

Logo Nutri-Score / Santé publique France 2017

Health Star Rating system

**Figure S1. Front-of-pack nutrition labels tested in the present study**

**Figure S2. Percentage of participants having deteriorated or improved their food choices between the two labelling conditions (without and with FoPL), for the overall sample**

**Figure S3. Percentage of correct answers in the two labelling conditions (without and with FoPL), for the overall sample**

| **Countries** | **N** | **HSR** | | **MTL** | | **Nutri-Score** | | **Warning symbol** | |
| --- | --- | --- | --- | --- | --- | --- | --- | --- | --- |
|  |  | **OR (95% CI)** | **P^b^** | **OR (95% CI)** | **P^b^** | **OR (95% CI)** | **P^b^** | **OR (95% CI)** | **P^b^** |
| **Overall** | | | | | | | | | |
| All^c^ | 11401 | 1.07 [0.93-1.22] | 0.5 | 1.21 [1.06-1.39] | 0.02 | 1.36 [1.19-1.55] | 0.0001 | 1.08 [0.95-1.23] | 0.4 |
| Belgium | 915 | 0.87 [0.57-1.33] | 0.9 | 1.22 [0.80-1.87] | 0.9 | 1.06 [0.69-1.63] | 1.0 | 0.86 [0.56-1.32] | 0·9 |
| Bulgaria | 966 | 1.23 [0.77-1.97] | 0.9 | 0.99 [0.62-1.58] | 1.0 | 1.16 [0.73-1.85] | 0.9 | 1.09 [0.68-1.74] | 1.0 |
| Denmark | 861 | 1.01 [0.58-1.74] | 1.0 | 1.15 [0.67-1.97] | 1.0 | 1.60 [0.95-2.72] | 0.5 | 1.07 [0.62-1.85] | 1.0 |
| France | 932 | 1.41 [0.91-2.18] | 0.6 | 1.57 [1.02-2.42] | 0.4 | 2.40 [1.55-3.71] | 0.02 | 1.70 [1.10-2.63] | 0.2 |
| Germany | 898 | 0.98 [0.60-1.60] | 1.0 | 0.97 [0.60-1.58] | 1.0 | 1.17 [0.72-1.89] | 0.9 | 0.72 [0.44-1.18] | 0.7 |
| Italy | 984 | 0.87 [0.57-1.32] | 0.9 | 0.97 [0.64-1.48] | 1.0 | 1.01 [0.67-1.54] | 1.0 | 0.92 [0.60-1.41] | 1.0 |
| Netherlands | 898 | 1.04 [0.65-1.66] | 1.0 | 0.90 [0.56-1.44] | 1.0 | 1.14 [0.72-1.81] | 1.0 | 0.99 [0.62-1.58] | 1.0 |
| Poland | 1081 | 0.94 [0.63-1.42] | 1.0 | 0.96 [0.64-1.45] | 1.0 | 0.87 [0.58-1.31] | 0.9 | 1.11 [0.74-1.67] | 1.0 |
| Portugal | 1007 | 1.20 [0.76-1.90] | 0.9 | 1.99 [1.27-3.12] | 0.1 | 1.94 [1.23-3.05] | 0.1 | 1.50 [0.95-2.37] | 0.5 |
| Spain | 951 | 0.86 [0.55-1.34] | 0.9 | 1.43 [0.92-2.22] | 0.5 | 1.25 [0.80-1.95] | 0.9 | 1.40 [0.91-2.17] | 0.6 |
| Switzerland | 1000 | 1.39 [0.88-2.19] | 0.6 | 1.17 [0.74-1.87] | 0.9 | 1.87 [1.20-2.92] | 0.1 | 0.81 [0.51-1.31] | 0.9 |
| United Kingdom | 908 | 1.12 [0.69-1.82] | 1.0 | 1.98 [1.23-3.20] | 0.1 | 1.80 [1.12-2.92] | 0.2 | 1.11 [0.69-1.79] | 1.0 |
| **Pizzas** | | | | | | | | | |
| All^c^ | 9597 | 1.07 [0.90-1.27] | 0.7 | 1.20 [1.01-1.43] | 0.2 | 1.28 [1.08-1.52] | 0.04 | 1.11 [0.94-1.32] | 0.6 |
| Belgium | 753 | 1.05 [0.61-1.82] | 1.0 | 1.18 [0.68-2.02] | 0.9 | 0.98 [0.57-1.68] | 1.0 | 0.86 [0.50-1.48] | 0.9 |
| Bulgaria | 846 | 1.23 [0.65-2.33] | 0.9 | 1.23 [0.65-2.30] | 0.9 | 0.83 [0.44-1.57] | 0.9 | 1.37 [0.73-2.58] | 0.8 |
| Denmark | 607 | 0.80 [0.38-1.65] | 0.9 | 1.37 [0.67-2.82] | 0.8 | 1.64 [0.82-3.28] | 0.7 | 1.00 [0.49-2.07] | 1.0 |
| France | 843 | 1.70 [1.00-2.88] | 0.6 | 1.38 [0.82-2.34] | 0.8 | 1.71 [1.01-2.89] | 0.6 | 1.31 [0.78-2.21] | 0.8 |
| Germany | 752 | 1.31 [0.67-2.56] | 0.8 | 1.50 [0.78-2.88] | 0.8 | 1.84 [0.96-3.52] | 0.6 | 1.15 [0.60-2.22] | 0.9 |
| Italy | 848 | 0.74 [0.43-1.27] | 0.8 | 0.81 [0.48-1.38] | 0.8 | 0.82 [0.48-1.39] | 0.8 | 0.80 [0.46-1.39] | 0.8 |
| Netherlands | 692 | 0.98 [0.52-1.86] | 1.0 | 0.84 [0.44-1.60] | 0.9 | 0.78 [0.42-1.48] | 0.8 | 0.69 [0.36-1.34] | 0.8 |
| Poland | 954 | 0.87 [0.52-1.47] | 0.9 | 1.01 [0.60-1.71] | 1.0 | 0.76 [0.45-1.29] | 0.8 | 1.36 [0.81-2.28] | 0.8 |
| Portugal | 864 | 0·83 [0.45-1.53] | 0.9 | 1.57 [0.88-2.81] | 0.7 | 1.87 [1.05-3.33] | 0.6 | 1.54 [0.86-2.76] | 0.7 |
| Spain | 866 | 0.89 [0.49-1.60] | 0.9 | 1.17 [0.65-2.09] | 0.9 | 1.55 [0.88-2.74] | 0.7 | 1.33 [0.75-2.35] | 0.8 |
| Switzerland | 834 | 1.52 [0.81-2.88] | 0.8 | 1.14 [0.60-2.19] | 0.9 | 1.98 [1.06-3.71] | 0.6 | 0.96 [0.50-1.86] | 1.0 |
| United Kingdom | 738 | 1.27 [0.66-2.44] | 0.8 | 2.10 [1.11-3.96] | 0.6 | 2.39 [1.26-4.53] | 0.5 | 1.20 [0.63-2.29] | 0.9 |
| **Cakes** | | | | | | | | | |
| All^c^ | 9355 | 0.96 [0.80-1.14] | 0.8 | 1.22 [1.02-1.45] | 0.1 | 1.25 [1.05-1.49] | 0.08 | 0.96 [0.80-1.15] | 0.8 |
| Belgium | 779 | 0.84 [0.48-1.44] | 0.9 | 1.55 [0.90-2.67] | 0.6 | 1.56 [0.91-2.67] | 0.5 | 0.90 [0.52-1.54] | 0.9 |
| Bulgaria | 832 | 0.75 [0.38-1.49] | 0.8 | 0.80 [0.40-1.58] | 0.9 | 1.03 [0.52-2.04] | 1.0 | 0.69 [0.34-1.41] | 0.8 |
| Denmark | 592 | 1.18 [0.54-2.58] | 0.9 | 1.19 [0.55-2.57] | 0.9 | 1.62 [0.77-3.42] | 0.7 | 0.88 [0.41-1.89] | 0.9 |
| France | 820 | 0.85 [0.50-1.44] | 0.9 | 1.22 [0.72-2.05] | 0.8 | 2.08 [1.23-3.52] | 0.1 | 1.30 [0.77-2.21] | 0.8 |
| Germany | 784 | 0.82 [0.40-1.66] | 0.9 | 0.74 [0.36-1.49] | 0.8 | 0.61 [0.30-1.26] | 0.7 | 0.64 [0.31-1.30] | 0.7 |
| Italy | 817 | 0.86 [0.50-1.50] | 0.9 | 0.86 [0.49-1.50] | 0.9 | 0.91 [0.52-1.60] | 0.9 | 1.01 [0.57-1.77] | 1.0 |
| Netherlands | 744 | 0.75 [0.41-1.37] | 0.8 | 0.87 [0.48-1.56] | 0.9 | 1.11 [0.62-1.99] | 0.9 | 0.77 [0.43-1.41] | 0.8 |
| Poland | 922 | 0.72 [0.43-1.21] | 0.7 | 0.78 [0.47-1.31] | 0.8 | 0.78 [0.47-1.31] | 0.8 | 0.97 [0.58-1.62] | 1.0 |
| Portugal | 759 | 1.42 [0.76-2.64] | 0.8 | 1.98 [1.08-3.65] | 0.3 | 2.25 [1.23-4.12] | 0.2 | 1.20 [0.63-2.25] | 0.9 |
| Spain | 772 | 0.93 [0.49-1.77] | 0.9 | 2.32 [1.25-4.28] | 0.1 | 1.16 [0.61-2.20] | 0.9 | 1.49 [0.79-2.82] | 0.7 |
| Switzerland | 781 | 1.37 [0.72-2.60] | 0.8 | 1.74 [0.92-3.26] | 0.5 | 1.70 [0.91-3.18] | 0.5 | 1.13 [0.58-2.22] | 0.9 |
| United Kingdom | 753 | 0.97 [0.49-1.90] | 1.0 | 1.64 [0.86-3.15] | 0.6 | 0.96 [0.49-1.89] | 1.0 | 0.58 [0.30-1.15] | 0.6 |
| **Breakfast cereals** | | | | | | | | | |
| All^c^ | 9340 | 1.15 [0.95-1.40] | 0.3 | 1.15 [0.95-1.40] | 0.3 | 1.37 [1.13-1.65] | 0.01 | 1.16 [0.95-1.40] | 0.3 |
| Belgium | 708 | 0.88 [0.50-1.57] | 0.9 | 0.83 [0.47-1.47] | 0.9 | 0.92 [0.52-1.63] | 1.0 | 0.90 [0.51-1.58] | 1.0 |
| Bulgaria | 780 | 1.31 [0.70-2.48] | 0.9 | 0.86 [0.45-1.64] | 0.9 | 1.17 [0.61-2.22] | 0.9 | 0.66 [0.34-1.29] | 0.7 |
| Denmark | 674 | 0.98 [0.40-2.40] | 1.0 | 0.74 [0.30-1.79] | 0.9 | 1.42 [0.59-3.42] | 0.9 | 1.83 [0.76-4.39] | 0.7 |
| France | 749 | 1.03 [0.58-1.84] | 1.0 | 1.18 [0.66-2.08] | 0.9 | 1.48 [0.84-2.63] | 0.7 | 1.58 [0.90-2.79] | 0.7 |
| Germany | 672 | 1.02 [0.52-2.00] | 1.0 | 1.06 [0.54-2.07] | 1.0 | 1.34 [0.68-2.62] | 0.9 | 0.69 [0.35-1.37] | 0.8 |
| Italy | 862 | 1.18 [0.64-2.17] | 0.9 | 1.47 [0.80-2.68] | 0.7 | 1.52 [0.83-2.77] | 0.7 | 0.97 [0.52-1.80] | 1.0 |
| Netherlands | 643 | 1.50 [0.74-3.03] | 0.8 | 0.87 [0.43-1.76] | 1.0 | 1.79 [0.88-3.61] | 0.7 | 2.26 [1.11-4.59] | 0.5 |
| Poland | 971 | 1.23 [0.66-2.29] | 0.9 | 1.29 [0.70-2.40] | 0.9 | 1.00 [0.54-1.87] | 1.0 | 1.09 [0.58-2.08] | 1.0 |
| Portugal | 890 | 1.62 [0.78-3.37] | 0.7 | 1.91 [0.91-3.98] | 0.7 | 2.02 [0.97-4.18] | 0.7 | 1.34 [0·63-2.86] | 0.9 |
| Spain | 833 | 0.88 [0.47-1.65] | 1.0 | 1.53 [0.82-2.87] | 0.7 | 0.98 [0.52-1.83] | 1.0 | 1.52 [0.82-2.82] | 0.7 |
| Switzerland | 779 | 1.48 [0.73-2.99] | 0.8 | 0.93 [0.46-1.89] | 1.0 | 1.58 [0.80-3.12] | 0.7 | 0.73 [0.36-1.50] | 0.9 |
| United Kingdom | 779 | 1.33 [0.62-2.87] | 0.9 | 2.25 [1.03-4.93] | 0.6 | 3.32 [1.52-7.25] | 0.1 | 2.46 [1.13-5.36] | 0.5 |

**Table S1. Associations^a^ between FoPLs and the change in nutritional quality of food choices, across and within the three food categories**

^a^ The reference of the multivariate ordinal logistic regression for the categorical variable ‘FoPL’ was the Reference Intakes label. The multivariate model was adjusted on sex, age, educational level, level of income, responsibility for grocery shopping, self-estimated diet quality, and self-estimated nutrition knowledge level.

^b^ P-values were obtained after a False Discovery Rate correction to consider multiple testing.

^c^ The model on the overall sample was conducted using a random effect on the FoPL variable.

HSR: Health Star Rating system; MTL: Multiple Traffic Lights; OR: Odds Ratio; CI: Confidence Interval.

**Table S2. Effect sizes of the associations between FoPLs and the change in nutritional quality of food choices, across and within the three food categories**

| **Countries** | **N** | **HSR** | | **MTL** | | **Nutri-Score** | | **Warning symbol** | |
| --- | --- | --- | --- | --- | --- | --- | --- | --- | --- |
|  |  | **OR (95% CI)** | **P^b^** | **OR (95% CI)** | **P^b^** | **OR (95% CI)** | **P^b^** | **OR (95% CI)** | **P^b^** |
| **Overall** | | | | | | | | | |
| All^c^ | 11401 | 0.04 (0.04) | 0.5 | 0.11 (0.04) | 0.02 | 0.17 (0.04) | 0.0001 | 0.04 (0.04) | 0.4 |
| Belgium | 915 | -0.08 (0.12) | 0.9 | 0.11 (0.12) | 0.9 | 0.03 (0.12) | 1.0 | -0.08 (0.12) | 0·9 |
| Bulgaria | 966 | 0.11 (0.13) | 0.9 | -0.01 (0.13) | 1.0 | 0.08 (0.13) | 0.9 | 0.05 (0.13) | 1.0 |
| Denmark | 861 | 0.01 (0.16) | 1.0 | 0.08 (0.15) | 1.0 | 0.26 (0.15) | 0.5 | 0.04 (0.15) | 1.0 |
| France | 932 | 0.19 (0.12) | 0.6 | 0.25 (0.12) | 0.4 | 0.48 (0.12) | 0.02 | 0.29 (0.12) | 0.2 |
| Germany | 898 | -0.01 (0.14) | 1.0 | -0.02 (0.14) | 1.0 | 0.09 (0.14) | 0.9 | -0.18 (0.14) | 0.7 |
| Italy | 984 | -0.08 (0.12) | 0.9 | -0.02 (0.12) | 1.0 | 0.01 (0.12) | 1.0 | -0.05 (0.12) | 1.0 |
| Netherlands | 898 | 0.02 (0.13) | 1.0 | -0.06 (0.13) | 1.0 | 0.07 (0.13) | 1.0 | -0.01 (0.13) | 1.0 |
| Poland | 1081 | -0.03 (0.11) | 1.0 | -0.02 (0.12) | 1.0 | -0.08 (0.11) | 0.9 | 0.06 (0.11) | 1.0 |
| Portugal | 1007 | 0.10 (0.13) | 0.9 | 0.38 (0.13) | 0.1 | 0.37 (0.13) | 0.1 | 0.22 (0.13) | 0.5 |
| Spain | 951 | -0.08 (0.13) | 0.9 | 0.20 (0.13) | 0.5 | 0.12 (0.13) | 0.9 | 0.19 (0.12) | 0.6 |
| Switzerland | 1000 | 0.18 (0.13) | 0.6 | 0.09 (0.13) | 0.9 | 0.35 (0.13) | 0.1 | -0.12 (0.13) | 0.9 |
| United Kingdom | 908 | 0.06 (0.14) | 1.0 | 0.38 (0.14) | 0.1 | 0.32 (0.14) | 0.2 | 0.06 (0.14) | 1.0 |
| **Pizzas** | | | | | | | | | |
| All^c^ | 9597 | 0.04 (0.05) | 0.7 | 0.10 (0.05) | 0.2 | 0.14 (0.05) | 0.04 | 0.06 (0.05) | 0.6 |
| Belgium | 753 | 0.03 (0.15) | 1.0 | 0.09 (0.15) | 0.9 | -0.01 (0.15) | 1.0 | -0.08 (0.15) | 0.9 |
| Bulgaria | 846 | 0.11 (0.18) | 0.9 | 0.11 (0.18) | 0.9 | -0.10 (0.18) | 0.9 | 0.17 (0.18) | 0.8 |
| Denmark | 607 | -0.12 (0.21) | 0.9 | 0.17 (0.20) | 0.8 | 0.27 (0.20) | 0.7 | 0.00 (0.20) | 1.0 |
| France | 843 | 0.29 (0.15) | 0.6 | 0.18 (0.15) | 0.8 | 0.30 (0.15) | 0.6 | 0.15 (0.15) | 0.8 |
| Germany | 752 | 0.15 (0.19) | 0.8 | 0.22 (0.18) | 0.8 | 0.34 (0.18) | 0.6 | 0.08 (0.18) | 0.9 |
| Italy | 848 | -0.17 (0.15) | 0.8 | -0.12 (0.15) | 0.8 | -0.11 (0.15) | 0.8 | -0.12 (0.16) | 0.8 |
| Netherlands | 692 | -0.01 (0.18) | 1.0 | -0.10 (0.18) | 0.9 | -0.14 (0.18) | 0.8 | -0.21 (0.18) | 0.8 |
| Poland | 954 | -0.08 (0.15) | 0.9 | 0.01 (0.15) | 1.0 | -0.15 (0.15) | 0.8 | 0.17 (0.15) | 0.8 |
| Portugal | 864 | -0.10 (0.17) | 0.9 | 0.25 (0.16) | 0.7 | 0.35 (0.16) | 0.6 | 0.24 (0.16) | 0.7 |
| Spain | 866 | -0.06 (0.17) | 0.9 | 0.09 (0.17) | 0.9 | 0.24 (0.16) | 0.7 | 0.16 (0.16) | 0.8 |
| Switzerland | 834 | 0.23 (0.18) | 0.8 | 0.07 (0.18) | 0.9 | 0.38 (0.18) | 0.6 | -0.02 (0.18) | 1.0 |
| United Kingdom | 738 | 0.13 (0.18) | 0.8 | 0.41 (0.18) | 0.6 | 0.48 (0.18) | 0.5 | 0.10 (0.18) | 0.9 |
| **Cakes** | | | | | | | | | |
| All^c^ | 9355 | -0.02 (0.05) | 0.8 | 0.11 (0.05) | 0.1 | 0.12 (0.05) | 0.08 | -0.02 (0.05) | 0.8 |
| Belgium | 779 | -0.10 (0.16) | 0.9 | 0.24 (0.15) | 0.6 | 0.25 (0.15) | 0.5 | -0.06 (0.15) | 0.9 |
| Bulgaria | 832 | -0.16 (0.19) | 0.8 | -0.12 (0.19) | 0.9 | 0.02 (0.19) | 1.0 | -0.21 (0.20) | 0.8 |
| Denmark | 592 | 0.09 (0.22) | 0.9 | 0.10 (0.22) | 0.9 | 0.27 (0.21) | 0.7 | -0.07 (0.21) | 0.9 |
| France | 820 | -0.09 (0.15) | 0.9 | 0.11 (0.15) | 0.8 | 0.40 (0.15) | 0.1 | 0.14 (0.15) | 0.8 |
| Germany | 784 | -0.11 (0.20) | 0.9 | -0.17 (0.20) | 0.8 | -0.27 (0.20) | 0.7 | -0.25 (0.20) | 0.7 |
| Italy | 817 | -0.08 (0.15) | 0.9 | -0.08 (0.16) | 0.9 | -0.05 (0.16) | 0.9 | 0.01 (0.16) | 1.0 |
| Netherlands | 744 | -0.16 (0.17) | 0.8 | -0.08 (0.17) | 0.9 | 0.06 (0.16) | 0.9 | -0.14 (0.17) | 0.8 |
| Poland | 922 | -0.18 (0.15) | 0.7 | -0.14 (0.15) | 0.8 | -0.14 (0.15) | 0.8 | -0.02 (0.15) | 1.0 |
| Portugal | 759 | 0.19 (0.18) | 0.8 | 0.38 (0.17) | 0.3 | 0.45 (0.17) | 0.2 | 0.10 (0.18) | 0.9 |
| Spain | 772 | -0.04 (0.18) | 0.9 | 0.46 (0.17) | 0.1 | 0.08 (0.18) | 0.9 | 0.22 (0.18) | 0.7 |
| Switzerland | 781 | 0.17 (0.18) | 0.8 | 0.31 (0.18) | 0.5 | 0.29 (0.18) | 0.5 | 0.07 (0.19) | 0.9 |
| United Kingdom | 753 | -0.02 (0.19) | 1.0 | 0.27 (0.18) | 0.6 | -0.02 (0.19) | 1.0 | -0.30 (0.19) | 0.6 |
| **Breakfast cereals** | | | | | | | | | |
| All^c^ | 9340 | 0.08 (0.06) | 0.3 | 0.08 (0.06) | 0.3 | 0.17 (0.05) | 0.01 | 0.08 (0.06) | 0.3 |
| Belgium | 708 | -0.07 (0.16) | 0.9 | -0.10 (0.16) | 0.9 | -0.05 (0.16) | 1.0 | -0.06 (0.16) | 1.0 |
| Bulgaria | 780 | 0.15 (0.18) | 0.9 | -0.08 (0.18) | 0.9 | 0.09 (0.18) | 0.9 | -0.23 (0.19) | 0.7 |
| Denmark | 674 | -0.01 (0.25) | 1.0 | -0.17 (0.25) | 0.9 | 0.19 (0.25) | 0.9 | 0.33 (0.25) | 0.7 |
| France | 749 | 0.02 (0.16) | 1.0 | 0.09 (0.16) | 0.9 | 0.22 (0.16) | 0.7 | 0.25 (0.16) | 0.7 |
| Germany | 672 | 0.01 (0.19) | 1.0 | 0.03 (0.19) | 1.0 | 0.16 (0.19) | 0.9 | -0.21 (0.19) | 0.8 |
| Italy | 862 | 0.09 (0.17) | 0.9 | 0.21 (0.17) | 0.7 | 0.23 (0.17) | 0.7 | -0.02 (0.17) | 1.0 |
| Netherlands | 643 | 0.22 (0.20) | 0.8 | -0.08 (0.20) | 1.0 | 0.32 (0.20) | 0.7 | 0.45 (0.20) | 0.5 |
| Poland | 971 | 0.11 (0.18) | 0.9 | 0.14 (0.17) | 0.9 | 0.00 (0.18) | 1.0 | 0.05 (0.18) | 1.0 |
| Portugal | 890 | 0.27 (0.21) | 0.7 | 0.36 (0.21) | 0.7 | 0.39 (0.21) | 0.7 | 0.16 (0.21) | 0.9 |
| Spain | 833 | -0.07 (0.18) | 1.0 | 0.23 (0.18) | 0.7 | -0.01 (0.18) | 1.0 | 0.23 (0.17) | 0.7 |
| Switzerland | 779 | 0.22 (0.20) | 0.8 | -0.04 (0.20) | 1.0 | 0.25 (0.19) | 0.7 | -0.17 (0.20) | 0.9 |
| United Kingdom | 779 | 0.16 (0.21) | 0.9 | 0.45 (0.22) | 0.6 | 0.66 (0.22) | 0.1 | 0.50 (0.22) | 0.5 |

^a^ The reference of the multivariate ordinal logistic regression for the categorical variable ‘FoPL’ was the Reference Intakes label.

The multivariate model was adjusted on sex, age, educational level, level of income, responsibility for grocery shopping, self-estimated diet quality, and self-estimated nutrition knowledge level. Values correspond to the Effect Size (Standard Error). They were obtained from the odds ratio, using published methodology (Chinn 2000)

^b^ P-values were obtained after a False Discovery Rate correction to consider multiple testing.

^c^ The model on the overall sample was conducted using a random effect on the FoPL variable.

HSR: Health Star Rating system; MTL: Multiple Traffic Lights; SE: Standard Error.

**Table S3. Associations^a^ between FoPLs and the change in nutritional quality of food choices, across and within the three food categories, adjusted on the response to “Did you see the label during the survey?”**

| **Countries** | **N** | **HSR** | | **MTL** | | **Nutri-Score** | | **Warning symbol** | |
| --- | --- | --- | --- | --- | --- | --- | --- | --- | --- |
|  |  | **OR (95% CI)** | **P^b^** | **OR (95% CI)** | **P^b^** | **OR (95% CI)** | **P^b^** | **OR (95% CI)** | **P^b^** |
| **Overall** | | | | | | | | | |
| All^c^ | 11401 | 1.19 [1.04-1.35] | 0.03 | 1.21 [1.06-1.39] | 0.01 | 1·37 [1.20-1.56] | <0.0001 | 1.26 [1.10-1.44] | 0.004 |
| Belgium | 915 | 0.92 [0.60-1.42] | 0.9 | 1.18 [0.77-1.81] | 0.8 | 1.02 [0.66-1.56] | 1.0 | 0.97 [0.63-1.50] | 1.0 |
| Bulgaria | 966 | 1.32 [0.82-2.11] | 0.7 | 0.98 [0.62-1.57] | 1.0 | 1.10 [0.68-1.77] | 0.9 | 1.18 [0.74-1.89] | 0.9 |
| Denmark | 861 | 1.16 [0.67-2.01] | 0.9 | 1.22 [0.71-2.11] | 0.8 | 1.67 [0.98-2.84] | 0.3 | 1.39 [0.80-2.43] | 0.7 |
| France | 932 | 1.56 [1.01-2.42] | 0.3 | 1.57 [1.02-2.42] | 0.3 | 2.44 [1.58-3.77] | 0.004 | 1.91 [1.23-2.96] | 0.05 |
| Germany | 898 | 1.09 [0.66-1.78] | 0.9 | 0.97 [0.60-1.58] | 1.0 | 1.13 [0.70-1.84] | 0.9 | 0.86 [0.52-1.42] | 0.9 |
| Italy | 984 | 0.94 [0.61-1.45] | 0.9 | 0.99 [0.65-1.51] | 1.0 | 1.06 [0.70-1.61] | 0.9 | 1.03 [0.67-1.60] | 1.0 |
| Netherlands | 898 | 1.21 [0.76-1.94] | 0.8 | 0.94 [0.59-1.51] | 0.9 | 1.10 [0.69-1.75] | 0.9 | 1.32 [0.82-2.13] | 0.7 |
| Poland | 1081 | 1.01 [0.67-1.53] | 1.0 | 0.96 [0.64-1.44] | 1.0 | 0.93 [0.61-1.40] | 0.9 | 1.28 [0.84-1.95] | 0.7 |
| Portugal | 1007 | 1.46 [0.92-2.32] | 0.4 | 1.95 [1.24-3.06] | 0.06 | 1.98 [1.26-3.12] | 0.05 | 1.94 [1.22-3.08] | 0.06 |
| Spain | 951 | 1.02 [0.64-1.62] | 1.0 | 1.46 [0.94-2.27] | 0.4 | 1.39 [0.89-2.18] | 0.5 | 1.63 [1.04-2.54] | 0.2 |
| Switzerland | 1000 | 1.44 [0.91-2.28] | 0.4 | 1.18 [0.74-1.88] | 0.9 | 1.83 [1.17-2.86] | 0.08 | 0.89 [0.56-1.44] | 0.9 |
| United Kingdom | 908 | 1.29 [0.79-2.10] | 0.7 | 1.94 [1.20-3.13] | 0.07 | 1.87 [1.16-3.03] | 0.1 | 1.33 [0.82-2.18] | 0.7 |
| **Pizzas** | | | | | | | | | |
| All^c^ | 9597 | 1.16 [0.98-1.38] | 0.3 | 1.20 [1.01-1.43] | 0.1 | 1.29 [1.08-1.53] | 0.02 | 1.26 [1.06-1.50] | 0.05 |
| Belgium | 753 | 1.11 [0.64-1.92] | 0.9 | 1.16 [0.68-2.00] | 0.9 | 0.97 [0.56-1.66] | 1.0 | 0.92 [0.53-1.60] | 0.9 |
| Bulgaria | 846 | 1.27 [0.67-2.42] | 0.8 | 1.24 [0.66-2.32] | 0.9 | 0.81 [0.42-1.55] | 0.9 | 1.43 [0.76-2.71] | 0.7 |
| Denmark | 607 | 0.88 [0.42-1.84] | 0.9 | 1.39 [0.67-2.87] | 0.8 | 1.73 [0.86-3.49] | 0.6 | 1.28 [0.61-2.69] | 0.9 |
| France | 843 | 1.90 [1.11-3.25] | 0.3 | 1.38 [0.81-2.34] | 0.7 | 1.73 [1.02-2.93] | 0.5 | 1.49 [0.88-2.52] | 0.6 |
| Germany | 752 | 1.48 [0.76-2.91] | 0.7 | 1.51 [0.78-2.9] | 0.7 | 1.81 [0.94-3.48] | 0.5 | 1.44 [0.74-2.82] | 0.7 |
| Italy | 848 | 0.77 [0.45-1.33] | 0.8 | 0.81 [0.48-1.38] | 0.8 | 0.82 [0.48-1.40] | 0.8 | 0.83 [0.47-1.45] | 0.9 |
| Netherlands | 692 | 1.11 [0.58-2.1] | 0.9 | 0.85 [0.45-1.64] | 0.9 | 0.76 [0.40-1.44] | 0.8 | 0.88 [0.45-1.73] | 0.9 |
| Poland | 954 | 0.90 [0.53-1.53] | 0.9 | 1.00 [0.59-1.70] | 1.0 | 0.79 [0.47-1.35] | 0.8 | 1.51 [0.88-2.59] | 0.6 |
| Portugal | 864 | 1.00 [0.54-1.86] | 1.0 | 1.56 [0.87-2.81] | 0.6 | 1.92 [1.07-3.43] | 0.4 | 1.99 [1.10-3.60] | 0.4 |
| Spain | 866 | 1.02 [0.56-1.88] | 1.0 | 1.21 [0.67-2.16] | 0.9 | 1.70 [0.96-3.03] | 0.5 | 1.51 [0.84-2.72] | 0.6 |
| Switzerland | 834 | 1.56 [0.82-2.96] | 0.6 | 1.14 [0.60-2.19] | 0.9 | 1.90 [1.01-3.57] | 0.5 | 1.09 [0.56-2.12] | 0.9 |
| United Kingdom | 738 | 1.49 [0.77-2.89] | 0.7 | 2.05 [1.08-3.89] | 0.4 | 2.47 [1.30-4.69] | 0.2 | 1.43 [0.74-2.76] | 0.7 |
| **Cakes** | | | | | | | | | |
| All^c^ | 9355 | 1.05 [0.88-1.26] | 0.8 | 1.22 [1.03-1.46] | 0.07 | 1.25 [1.05-1.49] | 0.06 | 1.10 [0.92-1.32] | 0.5 |
| Belgium | 779 | 0.88 [0.51-1.52] | 0.8 | 1.51 [0.87-2.60] | 0.5 | 1.50 [0.88-2.56] | 0.5 | 1.00 [0.57-1.73] | 1.0 |
| Bulgaria | 832 | 0.82 [0.41-1.63] | 0.8 | 0.79 [0.40-1.57] | 0.8 | 0.95 [0.47-1.89] | 1.0 | 0.74 [0.36-1.50] | 0.7 |
| Denmark | 592 | 1.29 [0.59-2.84] | 0.8 | 1.27 [0.59-2.75] | 0.8 | 1.63 [0.77-3.45] | 0.6 | 1.05 [0.48-2.28] | 1.0 |
| France | 820 | 0.92 [0.54-1.56] | 0.9 | 1.18 [0.70-1.98] | 0.8 | 2.08 [1.23-3.52] | 0.1 | 1.44 [0.85-2.45] | 0.6 |
| Germany | 784 | 0.89 [0.44-1.81] | 0.9 | 0.73 [0.36-1.49] | 0.7 | 0.61 [0.30-1.25] | 0.6 | 0.73 [0.35-1.51] | 0.7 |
| Italy | 817 | 0.98 [0.56-1.71] | 1.0 | 0.89 [0.51-1.56] | 0.9 | 0.97 [0.55-1.72] | 1.0 | 1.21 [0.68-2.16] | 0.8 |
| Netherlands | 744 | 0.81 [0.44-1.49] | 0.8 | 0.90 [0.50-1.63] | 0.9 | 1.10 [0.61-1.98] | 0.9 | 0.93 [0.50-1.71] | 0.9 |
| Poland | 922 | 0.81 [0.48-1.37] | 0.7 | 0.79 [0.47-1.32] | 0.7 | 0.86 [0.51-1.44] | 0.8 | 1.18 [0.69-2.01] | 0.8 |
| Portugal | 759 | 1.64 [0.87-3.06] | 0.5 | 1.89 [1.02-3.49] | 0.3 | 2.20 [1.20-4.03] | 0.1 | 1.41 [0.75-2.68] | 0.7 |
| Spain | 772 | 1.07 [0.55-2.08] | 1.0 | 2.38 [1.28-4.40] | 0.1 | 1.27 [0.66-2.44] | 0.8 | 1.68 [0.88-3.21] | 0.5 |
| Switzerland | 781 | 1.41 [0.74-2.69] | 0.7 | 1.74 [0.92-3.27] | 0.4 | 1.62 [0.86-3.03] | 0.5 | 1.26 [0.64-2.50] | 0.8 |
| United Kingdom | 753 | 1.04 [0.53-2.06] | 1.0 | 1.65 [0.86-3.15] | 0.5 | 0.97 [0.49-1.92] | 1.0 | 0.64 [0.32-1.28] | 0.6 |
| **Breakfast cereals** | | | | | | | | | |
| All^c^ | 9340 | 1.23 [1.01-1.48] | 0.1 | 1.16 [0.96-1.39] | 0.3 | 1.37 [1.14-1.66] | 0.009 | 1.26 [1.04-1.53] | 0.1 |
| Belgium | 708 | 0.93 [0.52-1.66] | 1.0 | 0.82 [0.46-1.44] | 0.9 | 0.87 [0.49-1.55] | 0.9 | 0.96 [0.54-1.71] | 1.0 |
| Bulgaria | 780 | 1.40 [0.74-2.65] | 0.8 | 0.87 [0.45-1.65] | 0.9 | 1.13 [0.59-2.17] | 1.0 | 0.71 [0.36-1.38] | 0.8 |
| Denmark | 674 | 1.03 [0.42-2.54] | 1.0 | 0.74 [0.31-1.80] | 0.9 | 1.42 [0.59-3.43] | 0.9 | 2.03 [0.83-4.94] | 0.6 |
| France | 749 | 1.10 [0.61-1.97] | 1.0 | 1.19 [0.67-2.11] | 0.9 | 1.50 [0.85-2.66] | 0.7 | 1.70 [0.96-3.01] | 0.6 |
| Germany | 672 | 1.04 [0.53-2.06] | 1.0 | 1.09 [0.56-2.13] | 1.0 | 1.33 [0.68-2.60] | 0.9 | 0.74 [0.36-1.50] | 0.9 |
| Italy | 862 | 1.22 [0.65-2.28] | 0.9 | 1.49 [0.81-2.72] | 0.7 | 1.54 [0.84-2.83] | 0.7 | 1.01 [0.53-1.92] | 1.0 |
| Netherlands | 643 | 1.72 [0.84-3.50] | 0.6 | 0.93 [0.46-1.88] | 1.0 | 1.77 [0.87-3.60] | 0.6 | 2.99 [1.45-6.21] | 0.1 |
| Poland | 971 | 1.26 [0.67-2.36] | 0.9 | 1.28 [0.69-2.39] | 0.9 | 1.04 [0.55-1.95] | 1.0 | 1.19 [0.61-2.30] | 0.9 |
| Portugal | 890 | 1.81 [0.87-3.77] | 0.6 | 1.82 [0.87-3.80] | 0.6 | 2.00 [0.96-4.13] | 0.6 | 1.55 [0.72-3.34] | 0.7 |
| Spain | 833 | 0.91 [0.48-1.73] | 1.0 | 1.54 [0.82-2.89] | 0.7 | 1.00 [0.53-1.90] | 1.0 | 1.56 [0.83-2.95] | 0.7 |
| Switzerland | 779 | 1.49 [0.74-3.02] | 0.7 | 0.94 [0.46-1.90] | 1.0 | 1.57 [0.79-3.12] | 0.7 | 0.75 [0.36-1.54] | 0.9 |
| United Kingdom | 779 | 1.47 [0.68-3.20] | 0.8 | 2.17 [0.99-4.79] | 0.6 | 3.41 [1.56-7.47] | 0.1 | 2.81 [1.28-6.16] | 0.3 |

^a^ The reference of the multivariate ordinal logistic regression for the categorical variable ‘FoPL’ was the Reference Intakes label. The multivariate model was adjusted on sex, age, educational level, level of income, responsibility for grocery shopping, self-estimated diet quality, self-estimated nutrition knowledge level, and the response to the question “Did you see the label during the survey?”.

^b^ P-values were obtained after a False Discovery Rate correction to consider multiple testing.

^c^ The model on the overall sample was conducted using a random effect on the FoPL variable.

HSR: Health Star Rating system; MTL: Multiple Traffic Lights; OR: Odds Ratio; CI: Confidence Interval.

**Table S4. Associations^a^ between FoPLs and the change in nutritional quality of food choices, adjusted on food category purchasing frequency**

| **Food categories** | **N** | **HSR** | | **MTL** | | **Nutri-Score** | | **Warning symbol** | |
| --- | --- | --- | --- | --- | --- | --- | --- | --- | --- |
|  |  | **OR (95% CI)** | **P^b^** | **OR (95% CI)** | **P^b^** | **OR (95% CI)** | **P^b^** | **OR (95% CI)** | **P^b^** |
| **All countries^c^** | | | | | | | | | |
| Pizza | 9597 | 1.07 [0.90-1.27] | 0.7 | 1.20 [1.01-1.43] | 0.2 | 1.28 [1.08-1.52] | 0.04 | 1.11 [0.94-1.52] | 0.6 |
| Cakes | 9355 | 0.96 [0.80-1.14] | 0.8 | 1.22 [1.02-1.45] | 0.1 | 1.24 [1.04-1.48] | 0.09 | 0.96 [0.80-1.15] | 0.8 |
| Breakfast cereals | 9340 | 1.15 [0.95-1.39] | 0.3 | 1.15 [0.95-1.39] | 0.3 | 1.36 [1.13-1.65] | 0.01 | 1.15 [0.95-1.39] | 0.3 |

^a^ The reference of the multivariate ordinal logistic regression for the categorical variable ‘FoPL’ was the Reference Intakes label. The multivariate model was adjusted on sex, age, educational level, level of income, responsibility for grocery shopping, self-estimated diet quality, self-estimated nutrition knowledge level, and the food category purchasing frequency.

^b^ P-values were obtained after a False Discovery Rate correction to consider multiple testing.

^c^ The model on the overall sample was conducted using a random effect on the FoPL variable.

HSR: Health Star Rating system; MTL: Multiple Traffic Lights; OR: Odds Ratio; CI: Confidence Interval.

**Table S5. Associations^a^ between FoPLs and the change in participants’ ability to correctly rank the nutritional quality of foods, across and within the three food categories**

| **Countries** | **N** | **HSR** | | **MTL** | | **Nutri-Score** | | **Warning symbol** | |  |
| --- | --- | --- | --- | --- | --- | --- | --- | --- | --- | --- |
|  |  | **OR (95% CI)** | **P^b^** | **OR (95% CI)** | **P^b^** | **OR (95% CI)** | **P^b^** | **OR (95% CI)** | **P^b^** | |
| **Overall** | | | | | | | | | |  |
| All^c^ | 12391 | 1.33 [1.14-1.57] | 0.002 | 1.66 [1.41-1.95] | <0.0001 | 3.15 [2.68-3.71] | <0.0001 | 1.24 [1.06-1.45] | 0.02 | |
| Belgium | 1007 | 1.18 [0.81-1.73] | 0.7 | 2.19 [1.50-3.19] | 0.0009 | 3.27 [2.24-4.79] | <0.0001 | 0.91 [0.62-1.34] | 0.8 | |
| Bulgaria | 1013 | 2.06 [1.41-3.02] | 0.003 | 1.31 [0.90-1.92] | 0.5 | 2.30 [1.57-3.37] | 0.0004 | 1.34 [0.92-1.96] | 0.5 | |
| Denmark | 1000 | 1.20 [0.83-1.73] | 0.7 | 1.70 [1.17-2.46] | 0.05 | 2.49 [1.72-3.60] | <0.0001 | 1.12 [0.77-1.62] | 0.8 | |
| France | 1000 | 1.56 [1.06-2.29] | 0.1 | 2.35 [1.61-3.45] | 0.0003 | 4.38 [2.98-6.43] | <0.0001 | 1.59 [1.08-2.34] | 0.1 | |
| Germany | 1000 | 1.33 [0.90-1.95] | 0.5 | 2.44 [1.66-3.58] | 0.0001 | 2.93 [2.00-4.31] | <0.0001 | 1.18 [0.80-1.74] | 0.7 | |
| Italy | 1032 | 1.59 [1.09-2.32] | 0.1 | 1.01 [0.69-1.47] | 1.0 | 2.18 [1.50-3.17] | 0.0008 | 1.02 [0.70-1.49] | 1.0 | |
| Netherlands | 1032 | 1.12 [0.77-1.64] | 0.8 | 1.30 [0.90-1.90] | 0.5 | 3.63 [2.50-5.27] | <0.0001 | 1.09 [0.75-1.59] | 0.8 | |
| Poland | 1160 | 1.22 [0.85-1.73] | 0.6 | 0.90 [0.63-1.29] | 0.8 | 2.12 [1.49-3.02] | 0.0006 | 1.65 [1.16-2.34] | 0.05 | |
| Portugal | 1059 | 1.67 [1.15-2.42] | 0.06 | 2.09 [1.44-3.03] | 0.002 | 6.21 [4.27-9.04] | <0.0001 | 1.58 [1.09-2.29] | 0.1 | |
| Spain | 1000 | 0.82 [0.56-1.20] | 0.6 | 1.83 [1.25-2.67] | 0.02 | 3.12 [2.14-4.56] | <0.0001 | 1.20 [0.82-1.74] | 0.7 | |
| Switzerland | 1088 | 1.26 [0.88-1.80] | 0.6 | 2.01 [1.41-2.88] | 0.002 | 4.18 [2.92-5.97] | <0.0001 | 1.18 [0.83-1.70] | 0.7 | |
| United Kingdom | 1000 | 1.39 [0.95-2.03] | 0.4 | 2.07 [1.42-3.01] | 0.002 | 4.14 [2.84-6.03] | <0.0001 | 1.16 [0.80-1.70] | 0.7 | |
| **Pizzas** | | | | | | | | | |  |
| All^c^ | 11896 | 1.20 [1.03-1.39] | 0.1 | 1.33 [1.14-1.54] | 0.003 | 2.06 [1.77-2.39] | <0.0001 | 1.08 [0.93-1.26] | 0.6 | |
| Belgium | 952 | 1.02 [0.64-1.63] | 1.0 | 1.61 [1.02-2.54] | 0.3 | 1.98 [1.26-3.12] | 0.08 | 0.83 [0.52-1.33] | 0.8 | |
| Bulgaria | 960 | 1.81 [1.15-2.85] | 0.2 | 1.09 [0.70-1.71] | 0.9 | 2.43 [1.54-3.83] | 0.009 | 1.33 [0.85-2.09] | 0.6 | |
| Denmark | 905 | 1.06 [0.66-1.70] | 0.9 | 1.45 [0.91-2.32] | 0.5 | 1.31 [0.82-2.07] | 0.6 | 0.95 [0.59-1.51] | 0.9 | |
| France | 979 | 1.67 [1.05-2.68] | 0.2 | 1.91 [1.20-3.06] | 0.1 | 2.55 [1.60-4.07] | 0.009 | 1.79 [1.12-2.86] | 0.2 | |
| Germany | 979 | 1.15 [0.71-1.85] | 0.9 | 1.74 [1.09-2.78] | 0.2 | 2.04 [1.28-3.25] | 0.08 | 1.11 [0.69-1.80] | 0.9 | |
| Italy | 1022 | 1.31 [0.83-2.08] | 0.6 | 1.09 [0.68-1.73] | 0.9 | 1.75 [1.11-2.76] | 0.2 | 0.84 [0.52-1.33] | 0.8 | |
| Netherlands | 972 | 1.36 [0.85-2.19] | 0.6 | 1.18 [0.73-1.89] | 0.9 | 2.18 [1.37-3.45] | 0.04 | 0.99 [0.62-1.58] | 1.0 | |
| Poland | 1128 | 0.90 [0.59-1.38] | 0.9 | 0.65 [0.42-0.99] | 0.3 | 1.61 [1.06-2.43] | 0.2 | 1.30 [0.86-1.97] | 0.6 | |
| Portugal | 1031 | 1.27 [0.82-1.98] | 0.7 | 1.30 [0.84-2.02] | 0.6 | 3.64 [2.37-5.59] | <0.0001 | 1.17 [0.76-1.82] | 0.8 | |
| Spain | 994 | 0.76 [0.48-1.23] | 0.6 | 1.62 [1.02-2.56] | 0.3 | 1.97 [1.25-3.12] | 0.08 | 0.73 [0.45-1.17] | 0.5 | |
| Switzerland | 1034 | 1.32 [0.82-2.11] | 0.6 | 1.48 [0.93-2.37] | 0.4 | 2.42 [1.54-3.82] | 0.009 | 1.19 [0.74-1.91] | 0.8 | |
| United Kingdom | 940 | 1.14 [0.70-1.85] | 0.9 | 1.72 [1.07-2.76] | 0.2 | 1.96 [1.22-3.14] | 0.1 | 0.93 [0.57-1.51] | 0.9 | |
| **Cakes** | | | | | | | | | |  |
| All^c^ | 12142 | 1.63 [1.35-1.96] | <0.0001 | 2.26 [1.87-2.73] | <0.0001 | 4.17 [3.46-5.02] | <0.0001 | 1.72 [1.43-2.08] | <0.0001 | |
| Belgium | 991 | 1.53 [0.94-2.48] | 0.3 | 2.57 [1.60-4.13] | 0.001 | 5.24 [3.29-8.35] | <0.0001 | 1.35 [0.83-2.20] | 0.6 | |
| Bulgaria | 1003 | 2.36 [1.46-3.82] | 0.006 | 1.62 [1.00-2.61] | 0.2 | 2.12 [1.31-3.42] | 0.02 | 1.46 [0.91-2.34] | 0.4 | |
| Denmark | 964 | 1.50 [0.96-2.34] | 0.3 | 2.43 [1.56-3.79] | 0.001 | 3.49 [2.26-5.40] | <0.0001 | 1.55 [1.00-2.41] | 0.3 | |
| France | 996 | 1.69 [1.03-2.76] | 0.2 | 3.70 [2.30-5.95] | <0.0001 | 7.57 [4.72-12.13] | <0.0001 | 1.81 [1.11-2.96] | 0.1 | |
| Germany | 976 | 2.12 [1.29-3.49] | 0.03 | 3.91 [2.40-6.38] | <0.0001 | 5.12 [3.15-8.34] | <0.0001 | 2.17 [1.31-3.59] | 0.02 | |
| Italy | 1028 | 1.57 [1.01-2.43] | 0.2 | 1.10 [0.71-1.71] | 0.9 | 2.07 [1.34-3.20] | 0.01 | 1.26 [0.81-1.96] | 0.7 | |
| Netherlands | 1019 | 1.31 [0.83-2.07] | 0.6 | 1.64 [1.04-2.58] | 0.2 | 4.44 [2.85-6.92] | <0.0001 | 1.78 [1.13-2.80] | 0.09 | |
| Poland | 1133 | 1.68 [1.09-2.60] | 0.1 | 1.17 [0.75-1.81] | 0.8 | 2.60 [1.69-4.00] | 0.0002 | 2.12 [1.38-3.28] | 0.008 | |
| Portugal | 1038 | 2.11 [1.37-3.26] | 0.008 | 3.17 [2.06-4.89] | <0.0001 | 6.86 [4.44-10.59] | <0.0001 | 2.24 [1.45-3.45] | 0.004 | |
| Spain | 975 | 1.04 [0.65-1.65] | 1.0 | 2.91 [1.85-4.58] | <0.0001 | 4.16 [2.65-6.54] | <0.0001 | 2.07 [1.31-3.25] | 0.02 | |
| Switzerland | 1039 | 1.45 [0.94-2.25] | 0.3 | 3.01 [1.96-4.61] | <0.0001 | 6.14 [4.02-9.38] | <0.0001 | 1.67 [1.08-2.58] | 0.1 | |
| United Kingdom | 980 | 1.86 [1.16-2.98] | 0.07 | 2.49 [1.56-3.98] | 0.002 | 6.48 [4.10-10.24] | <0.0001 | 1.93 [1.20-3.10] | 0.05 | |
| **Breakfast cereals** | | | | | | | | | |  |
| All^c^ | 11595 | 1.17 [1.02-1.34] | 0.06 | 1.29 [1.13-1.48] | 0.002 | 2.19 [1.91-2.50] | <0.0001 | 1.01 [0.88-1.16] | 0.9 | |
| Belgium | 917 | 1.16 [0.72-1.89] | 0.9 | 1.36 [0.85-2.20] | 0.8 | 1.60 [0.99-2.58] | 0.6 | 0.79 [0.48-1.30] | 0.9 | |
| Bulgaria | 964 | 1.88 [1.13-3.14] | 0.2 | 1.59 [0.95-2.64] | 0.6 | 2.26 [1.36-3.77] | 0.05 | 1.32 [0.79-2.20] | 0.8 | |
| Denmark | 978 | 0.99 [0.63-1.55] | 1.0 | 1.17 [0.76-1.82] | 0.9 | 1.61 [1.04-2.49] | 0.4 | 0.88 [0.56-1.37] | 0.9 | |
| France | 892 | 0.94 [0.58-1.53] | 1.0 | 1.21 [0.75-1.95] | 0.9 | 1.95 [1.22-3.12] | 0.1 | 1.06 [0.66-1.70] | 1.0 | |
| Germany | 879 | 1.30 [0.79-2.14] | 0.9 | 1.55 [0.95-2.52] | 0.6 | 2.18 [1.34-3.52] | 0.05 | 0.91 [0.55-1.51] | 1.0 | |
| Italy | 963 | 1.77 [1.08-2.90] | 0.3 | 1.02 [0.62-1.68] | 1.0 | 2.56 [1.58-4.14] | 0.006 | 1.06 [0.65-1.74] | 1.0 | |
| Netherlands | 931 | 0.86 [0.53-1.39] | 0.9 | 1.00 [0.62-1.61] | 1.0 | 2.68 [1.70-4.22] | 0.002 | 0.75 [0.46-1.22] | 0.8 | |
| Poland | 1115 | 1.44 [0.93-2.23] | 0.7 | 1.27 [0.82-1.98] | 0.8 | 1.93 [1.25-2.99] | 0.08 | 1.63 [1.05-2.54] | 0.4 | |
| Portugal | 1027 | 1.41 [0.87-2.27] | 0.8 | 1.79 [1.12-2.86] | 0.2 | 3.64 [2.31-5.73] | <0.0001 | 1.33 [0.82-2.14] | 0.8 | |
| Spain | 940 | 0.67 [0.42-1.07] | 0.7 | 0.94 [0.59-1.51] | 1.0 | 1.92 [1.23-2.98] | 0.08 | 0.89 [0.56-1.41] | 1.0 | |
| Switzerland | 1006 | 0.96 [0.61-1.49] | 1.0 | 1.32 [0.85-2.03] | 0.8 | 2.37 [1.56-3.61] | 0.003 | 0.86 [0.55-1.35] | 0.9 | |
| United Kingdom | 983 | 1.34 [0.85-2.09] | 0.8 | 1.71 [1.10-2.66] | 0.2 | 2.58 [1.67-3.98] | 0.002 | 0.86 [0.55-1.37] | 0.9 | |

^a^ The reference of the multivariate ordinal logistic regression for the categorical variable ‘FoPL’ was the Reference Intakes label.

The multivariate model was adjusted on sex, age, educational level, level of income, responsibility for grocery shopping, self-estimated diet quality, and self-estimated nutrition knowledge level.

^b^ P-values were obtained after a False Discovery Rate correction to consider multiple testing.

^c^ The model on the overall sample was conducted using a random effect on the FoPL variable.

HSR: Health Star Rating system; MTL: Multiple Traffic Lights; OR: Odds Ratio; CI: Confidence Interval.

**Table S6. Effect sizes of the associations between FoPLs and the change in participants’ ability to correctly rank the nutritional quality of foods, across and within the three food categories**

| **Countries** | **N** | **HSR** | | **MTL** | | **Nutri-Score** | | **Warning symbol** | |  |
| --- | --- | --- | --- | --- | --- | --- | --- | --- | --- | --- |
|  |  | **Effect size (SE)** | **P^b^** | **Effect size (SE)** | **P^b^** | **Effect size (SE)** | **P^b^** | **Effect size (SE)** | **P^b^** | |
| **Overall** | | | | | | | | | |  |
| All^c^ | 12391 | 0.16 (0.05) | 0.002 | 0.28 (0.05) | <0.0001 | 0.63 (0.05) | <0.0001 | 0.12 (0.05) | 0.02 | |
| Belgium | 1007 | 0.09 (0.11) | 0.7 | 0.43 (0.11) | 0.0009 | 0.65 (0.11) | <0.0001 | -0.05 (0.11) | 0.8 | |
| Bulgaria | 1013 | 0.40 (0.11) | 0.003 | 0.15 (0.11) | 0.5 | 0.46 (0.11) | 0.0004 | 0.16 (0.11) | 0.5 | |
| Denmark | 1000 | 0.10 (0.10) | 0.7 | 0.29 (0.10) | 0.05 | 0.50 (0.10) | <0.0001 | 0.06 (0.10) | 0.8 | |
| France | 1000 | 0.25 (0.11) | 0.1 | 0.47 (0.11) | 0.0003 | 0.82 (0.11) | <0.0001 | 0.26 (0.11) | 0.1 | |
| Germany | 1000 | 0.16 (0.11) | 0.5 | 0.49 (0.11) | 0.0001 | 0.59 (0.11) | <0.0001 | 0.09 (0.11) | 0.7 | |
| Italy | 1032 | 0.26 (0.10) | 0.1 | 0.01 (0.11) | 1.0 | 0.43 (0.11) | 0.0008 | 0.01 (0.11) | 1.0 | |
| Netherlands | 1032 | 0.06 (0.10) | 0.8 | 0.14 (0.10) | 0.5 | 0.71 (0.10) | <0.0001 | 0.05 (0.11) | 0.8 | |
| Poland | 1160 | 0.11 (0.10) | 0.6 | -0.06 (0.10) | 0.8 | 0.42 (0.10) | 0.0006 | 0.28 (0.10) | 0.05 | |
| Portugal | 1059 | 0.28 (0.10) | 0.06 | 0.41 (0.10) | 0.002 | 1.01 (0.11) | <0.0001 | 0.25 (0.10) | 0.1 | |
| Spain | 1000 | -0.11 (0.11) | 0.6 | 0.33 (0.11) | 0.02 | 0.63 (0.11) | <0.0001 | 0.1 (0.11) | 0.7 | |
| Switzerland | 1088 | 0.13 (0.10) | 0.6 | 0.39 (0.10) | 0.002 | 0.79 (0.10) | <0.0001 | 0.09 (0.10) | 0.7 | |
| United Kingdom | 1000 | 0.18 (0.11) | 0.4 | 0.40 (0.11) | 0.002 | 0.78 (0.10) | <0.0001 | 0.08 (0.10) | 0.7 | |
| **Pizzas** | | | | | | | | | |  |
| All^c^ | 11896 | 0.10 (0.04) | 0.1 | 0.16 (0.04) | 0.003 | 0.40 (0.04) | <0.0001 | 0.04 (0.04) | 0.6 | |
| Belgium | 952 | 0.01 (0.13) | 1.0 | 0.26 (0.13) | 0.3 | 0.38 (0.13) | 0.08 | -0.1 (0.13) | 0.8 | |
| Bulgaria | 960 | 0.33 (0.13) | 0.2 | 0.05 (0.13) | 0.9 | 0.49 (0.13) | 0.009 | 0.16 (0.13) | 0.6 | |
| Denmark | 905 | 0.03 (0.13) | 0.9 | 0.21 (0.13) | 0.5 | 0.15 (0.13) | 0.6 | -0.03 (0.13) | 0.9 | |
| France | 979 | 0.28 (0.13) | 0.2 | 0.36 (0.13) | 0.1 | 0.52 (0.13) | 0.009 | 0.32 (0.13) | 0.2 | |
| Germany | 979 | 0.08 (0.14) | 0.9 | 0.31 (0.13) | 0.2 | 0.39 (0.13) | 0.08 | 0.06 (0.14) | 0.9 | |
| Italy | 1022 | 0.15 (0.13) | 0.6 | 0.05 (0.13) | 0.9 | 0.31 (0.13) | 0.2 | -0.10 (0.13) | 0.8 | |
| Netherlands | 972 | 0.17 (0.13) | 0.6 | 0.09 (0.13) | 0.9 | 0.43 (0.13) | 0.04 | -0.01 (0.13) | 1.0 | |
| Poland | 1128 | -0.06 (0.12) | 0.9 | -0.24 (0.12) | 0.3 | 0.26 (0.12) | 0.2 | 0.14 (0.11) | 0.6 | |
| Portugal | 1031 | 0.13 (0.13) | 0.7 | 0.14 (0.13) | 0.6 | 0.71 (0.12) | <0.0001 | 0.09 (0.12) | 0.8 | |
| Spain | 994 | -0.15 (0.13) | 0.6 | 0.27 (0.13) | 0.3 | 0.37 (0.13) | 0.08 | -0.17 (0.14) | 0.5 | |
| Switzerland | 1034 | 0.15 (0.13) | 0.6 | 0.22 (0.13) | 0.4 | 0.49 (0.13) | 0.009 | 0.10 (0.14) | 0.8 | |
| United Kingdom | 940 | 0.07 (0.14) | 0.9 | 0.30 (0.13) | 0.2 | 0.37 (0.13) | 0.1 | -0.04 (0.14) | 0.9 | |
| **Cakes** | | | | | | | | | |  |
| All^c^ | 12142 | 0.27 (0.05) | <0.0001 | 0.45 (0.05) | <0.0001 | 0.79 (0.05) | <0.0001 | 0.30 (0.05) | <0.0001 | |
| Belgium | 991 | 0.23 (0.14) | 0.3 | 0.52 (0.13) | 0.001 | 0.92 (0.13) | <0.0001 | 0.17 (0.14) | 0.6 | |
| Bulgaria | 1003 | 0.47 (0.14) | 0.006 | 0.27 (0.14) | 0.2 | 0.42 (0.14) | 0.02 | 0.21 (0.13) | 0.4 | |
| Denmark | 964 | 0.22 (0.13) | 0.3 | 0.49 (0.13) | 0.001 | 0.69 (0.12) | <0.0001 | 0.24 (0.13) | 0.3 | |
| France | 996 | 0.29 (0.14) | 0.2 | 0.72 (0.14) | <0.0001 | 1.12 (-0.11) | <0.0001 | 0.33 (0.14) | 0.1 | |
| Germany | 976 | 0.42 (0.14) | 0.03 | 0.75 (0.14) | <0.0001 | 0.90 (0.14) | <0.0001 | 0.43 (0.14) | 0.02 | |
| Italy | 1028 | 0.25 (0.12) | 0.2 | 0.05 (0.13) | 0.9 | 0.40 (0.12) | 0.01 | 0.13 (0.13) | 0.7 | |
| Netherlands | 1019 | 0.15 (0.13) | 0.6 | 0.27 (0.13) | 0.2 | 0.82 (0.13) | <0.0001 | 0.32 (0.13) | 0.09 | |
| Poland | 1133 | 0.29 (0.12) | 0.1 | 0.09 (0.13) | 0.8 | 0.53 (0.12) | 0.0002 | 0.42 (0.12) | 0.008 | |
| Portugal | 1038 | 0.41 (0.12) | 0.008 | 0.64 (0.12) | <0.0001 | 1.06 (-0.28) | <0.0001 | 0.45 (0.12) | 0.004 | |
| Spain | 975 | 0.02 (0.13) | 1.0 | 0.59 (0.13) | <0.0001 | 0.79 (0.13) | <0.0001 | 0.40 (0.13) | 0.02 | |
| Switzerland | 1039 | 0.21 (0.12) | 0.3 | 0.61 (0.12) | <0.0001 | 1.00 (0.12) | <0.0001 | 0.28 (0.12) | 0.1 | |
| United Kingdom | 980 | 0.34 (0.13) | 0.07 | 0.50 (0.13) | 0.002 | 1.03 (-0.4) | <0.0001 | 0.36 (0.14) | 0.05 | |
| **Breakfast cereals** | | | | | | | | | |  |
| All^c^ | 11595 | 0.09 (0.04) | 0.06 | 0.14 (0.04) | 0.002 | 0.43 (0.04) | <0.0001 | 0.01 (0.04) | 0.9 | |
| Belgium | 917 | 0.08 (0.14) | 0.9 | 0.17 (0.14) | 0.8 | 0.26 (0.14) | 0.6 | -0.13 (0.14) | 0.9 | |
| Bulgaria | 964 | 0.35 (0.14) | 0.2 | 0.26 (0.15) | 0.6 | 0.45 (0.14) | 0.05 | 0.15 (0.15) | 0.8 | |
| Denmark | 978 | -0.01 (0.13) | 1.0 | 0.09 (0.12) | 0.9 | 0.26 (0.12) | 0.4 | -0.07 (0.13) | 0.9 | |
| France | 892 | -0.03 (0.14) | 1.0 | 0.11 (0.14) | 0.9 | 0.37 (0.13) | 0.1 | 0.03 (0.13) | 1.0 | |
| Germany | 879 | 0.14 (0.14) | 0.9 | 0.24 (0.14) | 0.6 | 0.43 (0.14) | 0.05 | -0.05 (0.14) | 1.0 | |
| Italy | 963 | 0.32 (0.14) | 0.3 | 0.01 (0.14) | 1.0 | 0.52 (0.14) | 0.006 | 0.03 (0.14) | 1.0 | |
| Netherlands | 931 | -0.08 (0.14) | 0.9 | 0.00 (0.13) | 1.0 | 0.54 (0.13) | 0.002 | -0.16 (0.14) | 0.8 | |
| Poland | 1115 | 0.20 (0.12) | 0.7 | 0.13 (0.13) | 0.8 | 0.36 (0.13) | 0.08 | 0.27 (0.13) | 0.4 | |
| Portugal | 1027 | 0.19 (0.14) | 0.8 | 0.32 (0.13) | 0.2 | 0.71 (0.13) | <0.0001 | 0.16 (0.14) | 0.8 | |
| Spain | 940 | -0.22 (0.13) | 0.7 | -0.03 (0.13) | 1.0 | 0.36 (0.13) | 0.08 | -0.06 (0.13) | 1 | |
| Switzerland | 1006 | -0.02 (0.13) | 1.0 | 0.15 (0.12) | 0.8 | 0.48 (0.12) | 0.003 | -0.08 (0.13) | 0.9 | |
| United Kingdom | 983 | 0.16 (0.13) | 0.8 | 0.30 (0.13) | 0.2 | 0.52 (0.12) | 0.002 | -0.08 (0.13) | 0.9 | |

^a^ The reference of the multivariate ordinal logistic regression for the categorical variable ‘FoPL’ was the Reference Intakes label.

The multivariate model was adjusted on sex, age, educational level, level of income, responsibility for grocery shopping, self-estimated diet quality, and self-estimated nutrition knowledge level. Values correspond to the Effect Size (Standard Error). They were obtained from the odds ratio, using published methodology (Chinn 2000)

^b^ P-values were obtained after a False Discovery Rate correction to consider multiple testing.

^c^ The model on the overall sample was conducted using a random effect on the FoPL variable.

HSR: Health Star Rating system; MTL: Multiple Traffic Lights; SE: Standard Error.

**Table S7. Associations^a^ between FoPLs and the change in participants’ ability to correctly rank the nutritional quality of foods, across and within the three food categories, adjusted on the response to “Did you see the label during the survey?”**

| **Countries** | **N** | **HSR** | | **MTL** | | **Nutri-Score** | | **Warning symbol** | |
| --- | --- | --- | --- | --- | --- | --- | --- | --- | --- |
|  |  | **OR (95% CI)** | **P^b^** | **OR (95% CI)** | **P^b^** | **OR (95% CI)** | **P^b^** | **OR (95% CI)** | **P^b^** |
| **Overall** | | | | | | | | | |
| All^c^ | 12391 | 1.52 [1.29-1.80] | <0.0001 | 1.68 [1.42-1.98] | <0.0001 | 3.23 [2.75-3.81] | <0.0001 | 1.49 [1.27-1.76] | <0.0001 |
| Belgium | 1007 | 1.36 [0.93-2.00] | 0.3 | 2.06 [1.41-3.01] | 0.002 | 3.04 [2.07-4.45] | <0.0001 | 1.14 [0.77-1.69] | 0.7 |
| Bulgaria | 1013 | 1.95 [1.33-2.86] | 0.005 | 1.31 [0.90-1.91] | 0.4 | 2.46 [1.66-3.62] | <0.0001 | 1.28 [0.87-1.87] | 0.5 |
| Denmark | 1000 | 1.35 [0.93-1.96] | 0.3 | 1.79 [1.23-2.59] | 0.01 | 2.50 [1.73-3.61] | <0.0001 | 1.41 [0.97-2.06] | 0.3 |
| France | 1000 | 1.85 [1.26-2.73] | 0.01 | 2.33 [1.59-3.42] | 0.0002 | 4.69 [3.18-6.90] | <0.0001 | 1.92 [1.30-2.83] | 0.007 |
| Germany | 1000 | 1.73 [1.17-2.56] | 0.03 | 2.63 [1.79-3.88] | <0.0001 | 3.03 [2.06-4.46] | <0.0001 | 1.64 [1.10-2.45] | 0.07 |
| Italy | 1032 | 1.77 [1.20-2.59] | 0.02 | 1.06 [0.72-1.54] | 0.9 | 2.33 [1.60-3.40] | 0.0001 | 1.19 [0.80-1.75] | 0.6 |
| Netherlands | 1032 | 1.20 [0.82-1.75] | 0.6 | 1.31 [0.90-1.90] | 0.4 | 3.60 [2.48-5.24] | <0.0001 | 1.23 [0.84-1.81] | 0.5 |
| Poland | 1160 | 1.32 [0.92-1.89] | 0.4 | 0.90 [0.63-1.28] | 0.7 | 2.30 [1.61-3.28] | <0.0001 | 1.91 [1.32-2.75] | 0.004 |
| Portugal | 1059 | 2.02 [1.39-2.94] | 0.002 | 2.11 [1.46-3.05] | 0.0007 | 6.45 [4.43-9.39] | <0.0001 | 2.00 [1.37-2.91] | 0.003 |
| Spain | 1000 | 1.06 [0.72-1.57] | 0.9 | 1.94 [1.33-2.83] | 0.005 | 3.87 [2.63-5.70] | <0.0001 | 1.54 [1.04-2.26] | 0.1 |
| Switzerland | 1088 | 1.43 [1.00-2.05] | 0.2 | 2.09 [1.46-2.99] | 0.0005 | 4.02 [2.81-5.75] | <0.0001 | 1.52 [1.05-2.18] | 0.1 |
| United Kingdom | 1000 | 1.79 [1.22-2.63] | 0.02 | 2.08 [1.43-3.02] | 0.001 | 4.68 [3.20-6.84] | <0.0001 | 1.61 [1.09-2.37] | 0.07 |
| **Pizzas** | | | | | | | | | |
| All^c^ | 11896 | 1.28 [1.10-1.49] | 0.007 | 1.33 [1.14-1.54] | 0.002 | 2.08 [1.79-2.41] | <0.0001 | 1.19 [1.02-1.39] | 0.09 |
| Belgium | 952 | 1.13 [0.71-1.81] | 0.9 | 1.52 [0.96-2.41] | 0.3 | 1.84 [1.16-2.90] | 0.1 | 1.00 [0.62-1.61] | 1.0 |
| Bulgaria | 960 | 1.69 [1.07-2.66] | 0.2 | 1.10 [0.70-1.72] | 0.9 | 2.66 [1.68-4.24] | 0.003 | 1.26 [0.80-1.97] | 0.7 |
| Denmark | 905 | 1.17 [0.73-1.88] | 0.8 | 1.52 [0.95-2.42] | 0.3 | 1.31 [0.82-2.08] | 0.6 | 1.15 [0.71-1.87] | 0.8 |
| France | 979 | 1.86 [1.16-2.99] | 0.1 | 1.85 [1.16-2.96] | 0.1 | 2.53 [1.59-4.04] | 0.004 | 2.00 [1.24-3.21] | 0.05 |
| Germany | 979 | 1.33 [0.82-2.16] | 0.6 | 1.81 [1.13-2.90] | 0.1 | 1.99 [1.25-3.19] | 0.05 | 1.32 [0.81-2.16] | 0.6 |
| Italy | 1022 | 1.47 [0.92-2.34] | 0.4 | 1.12 [0.71-1.79] | 0.9 | 1.89 [1.20-2.99] | 0.07 | 0.97 [0.60-1.57] | 0.9 |
| Netherlands | 972 | 1.37 [0.85-2.21] | 0.5 | 1.17 [0.73-1.88] | 0.8 | 2.12 [1.34-3.37] | 0.03 | 1.00 [0.62-1.62] | 1.0 |
| Poland | 1128 | 0.94 [0.61-1.44] | 0.9 | 0.65 [0.42-0.99] | 0.2 | 1.64 [1.08-2.50] | 0.1 | 1.34 [0.87-2.06] | 0.5 |
| Portugal | 1031 | 1.42 [0.91-2.22] | 0.4 | 1.30 [0.84-2.01] | 0.6 | 3.67 [2.39-5.64] | <0.0001 | 1.33 [0.85-2.08] | 0.5 |
| Spain | 994 | 0.87 [0.53-1.42] | 0.8 | 1.66 [1.05-2.63] | 0.2 | 2.15 [1.36-3.42] | 0.03 | 0.81 [0.50-1.33] | 0.7 |
| Switzerland | 1034 | 1.43 [0.89-2.30] | 0.4 | 1.50 [0.94-2.40] | 0.3 | 2.36 [1.49-3.72] | 0.007 | 1.39 [0.86-2.26] | 0.5 |
| United Kingdom | 940 | 1.24 [0.76-2.02] | 0.7 | 1.69 [1.05-2.72] | 0.2 | 2.00 [1.25-3.22] | 0.05 | 1.03 [0.63-1.69] | 0.9 |
| **Cakes** | | | | | | | | | |
| All^c^ | 12142 | 1.85 [1.52-2.24] | <0.0001 | 2.28 [1.88-2.76] | <0.0001 | 4.28 [3.54-5.18] | <0.0001 | 2.06 [1.70-2.50] | <0.0001 |
| Belgium | 991 | 1.72 [1.06-2.80] | 0.1 | 2.46 [1.53-3.95] | 0.002 | 4.88 [3.06-7.78] | <0.0001 | 1.65 [1.01-2.70] | 0.2 |
| Bulgaria | 1003 | 2.28 [1.41-3.69] | 0.005 | 1.63 [1.01-2.63] | 0.2 | 2.23 [1.37-3.63] | 0.007 | 1.41 [0.88-2.27] | 0.4 |
| Denmark | 964 | 1.65 [1.06-2.59] | 0.1 | 2.58 [1.66-4.03] | 0.0003 | 3.60 [2.33-5.58] | <0.0001 | 1.88 [1.20-2.95] | 0.03 |
| France | 996 | 2.01 [1.23-3.31] | 0.03 | 3.64 [2.26-5.86] | <0.0001 | 8.18 [5.07-13.19] | <0.0001 | 2.19 [1.33-3.59] | 0.01 |
| Germany | 976 | 2.57 [1.56-4.25] | 0.002 | 4.10 [2.51-6.71] | <0.0001 | 5.17 [3.17-8.45] | <0.0001 | 2.84 [1.70-4.75] | 0.0006 |
| Italy | 1028 | 1.63 [1.04-2.54] | 0.1 | 1.13 [0.73-1.76] | 0.8 | 2.15 [1.39-3.33] | 0.004 | 1.37 [0.87-2.15] | 0.5 |
| Netherlands | 1019 | 1.42 [0.89-2.24] | 0.4 | 1.66 [1.05-2.62] | 0.1 | 4.52 [2.89-7.06] | <0.0001 | 2.10 [1.32-3.34] | 0.009 |
| Poland | 1133 | 1.78 [1.15-2.77] | 0.05 | 1.16 [0.74-1.80] | 0.8 | 2.86 [1.85-4.43] | <0.0001 | 2.60 [1.66-4.08] | 0.0003 |
| Portugal | 1038 | 2.56 [1.65-3.97] | 0.0003 | 3.19 [2.07-4.93] | <0.0001 | 7.14 [4.61-11.07] | <0.0001 | 2.88 [1.85-4.48] | <0.0001 |
| Spain | 975 | 1.42 [0.89-2.28] | 0.4 | 3.16 [2.00-4.99] | <0.0001 | 5.34 [3.36-8.50] | <0.0001 | 2.77 [1.74-4.43] | 0.0002 |
| Switzerland | 1039 | 1.64 [1.06-2.54] | 0.1 | 3.11 [2.03-4.78] | <0.0001 | 5.97 [3.90-9.15] | <0.0001 | 2.09 [1.35-3.25] | 0.006 |
| United Kingdom | 980 | 2.34 [1.45-3.78] | 0.003 | 2.45 [1.54-3.90] | 0.001 | 7.37 [4.64-11.7] | <0.0001 | 2.67 [1.65-4.32] | 0.0006 |
| **Breakfast cereals** | | | | | | | | | |
| All^c^ | 11595 | 1.31 [1.14-1.51] | 0.001 | 1.29 [1.12-1.48] | 0.002 | 2.22 [1.94-2.54] | <0.0001 | 1.19 [1.03-1.37] | 0.03 |
| Belgium | 917 | 1.32 [0.81-2.16] | 0.8 | 1.28 [0.79-2.07] | 0.8 | 1.52 [0.94-2.46] | 0.4 | 0.97 [0.58-1.60] | 1.0 |
| Bulgaria | 964 | 1.91 [1.14-3.19] | 0.1 | 1.57 [0.94-2.62] | 0.4 | 2.18 [1.30-3.66] | 0.04 | 1.32 [0.79-2.21] | 0.8 |
| Denmark | 978 | 1.08 [0.69-1.69] | 1.0 | 1.22 [0.79-1.9] | 0.8 | 1.60 [1.03-2.48] | 0.2 | 1.05 [0.66-1.67] | 1.0 |
| France | 892 | 1.03 [0.64-1.68] | 1.0 | 1.17 [0.73-1.89] | 0.9 | 1.97 [1.23-3.16] | 0.05 | 1.15 [0.71-1.85] | 0.9 |
| Germany | 879 | 1.6 [0.97-2.65] | 0.4 | 1.55 [0.95-2.53] | 0.4 | 2.21 [1.36-3.60] | 0.02 | 1.30 [0.78-2.19] | 0.8 |
| Italy | 963 | 1.97 [1.19-3.26] | 0.07 | 1.08 [0.66-1.78] | 1.0 | 2.76 [1.70-4.49] | 0.001 | 1.26 [0.76-2.10] | 0.8 |
| Netherlands | 931 | 0.90 [0.56-1.47] | 1.0 | 1.00 [0.62-1.62] | 1.0 | 2.66 [1.68-4.21] | 0.001 | 0.85 [0.52-1.39] | 0.9 |
| Poland | 1115 | 1.60 [1.03-2.50] | 0.2 | 1.26 [0.81-1.96] | 0.8 | 2.12 [1.36-3.29] | 0.02 | 1.93 [1.22-3.05] | 0.05 |
| Portugal | 1027 | 1.58 [0.98-2.56] | 0.4 | 1.77 [1.11-2.83] | 0.1 | 3.68 [2.33-5.80] | <0.0001 | 1.56 [0.96-2.53] | 0.4 |
| Spain | 940 | 0.81 [0.50-1.32] | 0.9 | 0.97 [0.61-1.55] | 1.0 | 2.21 [1.41-3.47] | 0.01 | 1.06 [0.67-1.70] | 1.0 |
| Switzerland | 1006 | 1.05 [0.68-1.64] | 1.0 | 1.29 [0.83-1.98] | 0.8 | 2.25 [1.47-3.43] | 0.004 | 1.03 [0.65-1.61] | 1.0 |
| United Kingdom | 983 | 1.71 [1.08-2.70] | 0.2 | 1.66 [1.07-2.59] | 0.2 | 2.86 [1.84-4.44] | 0.0002 | 1.20 [0.75-1.91] | 0.9 |

^a^ The reference of the multivariate ordinal logistic regression for the categorical variable ‘FoPL’ was the Reference Intakes label. The multivariate model was adjusted on sex, age, educational level, level of income, responsibility for grocery shopping, self-estimated diet quality, self-estimated nutrition knowledge level, and the response to the question “Did you see the label during the survey?”.

^b^ P-values were obtained after a False Discovery Rate correction to consider multiple testing.

^c^ The model on the overall sample was conducted using a random effect on the FoPL variable.

HSR: Health Star Rating system; MTL: Multiple Traffic Lights; OR: Odds Ratio; CI: Confidence Interval.

**Table S8. Associations^a^ between FoPLs and the change in participants’ ability to correctly rank the nutritional quality of foods, across and within the three food categories, with no distinction between non-response and incorrect ranking**

| **Countries** | **N** | **HSR** | | **MTL** | | **Nutri-Score** | | **Warning symbol** | |  |
| --- | --- | --- | --- | --- | --- | --- | --- | --- | --- | --- |
|  |  | **OR (95% CI)** | **P^b^** | **OR (95% CI)** | **P^b^** | **OR (95% CI)** | **P^b^** | **OR (95% CI)** | **P^b^** | |
| **Overall** | | | | | | | | | |  |
| All^c^ | 12391 | 1.30 [1.10-1.53] | 0.007 | 1.67 [1.42-1.96] | <0.0001 | 3.23 [2.75-3.81] | <0.0001 | 1.21 [1.03-1.43] | 0.05 |  |
| Belgium | 1007 | 1.14 [0.75-1.71] | 0.8 | 2.37 [1.58-3.53] | 0.0005 | 3.49 [2.34-5.21] | <0.0001 | 0.89 [0.59-1.34] | 0.8 |  |
| Bulgaria | 1013 | 1.97 [1.31-2.97] | 0.01 | 1.12 [0.74-1.67] | 0.8 | 2.34 [1.55-3.53] | 0.0009 | 1.28 [0.85-1.91] | 0.6 |  |
| Denmark | 1000 | 1.09 [0.75-1.60] | 0.8 | 1.65 [1.13-2.40] | 0.08 | 2.46 [1.69-3.58] | <0.0001 | 1.02 [0.70-1.49] | 1.0 |  |
| France | 1000 | 1.53 [1.03-2.27] | 0.2 | 2.42 [1.63-3.57] | 0.0002 | 4.29 [2.90-6.35] | <0.0001 | 1.51 [1.02-2.24] | 0.2 |  |
| Germany | 1000 | 1.20 [0.80-1.80] | 0.7 | 2.15 [1.44-3.21] | 0.003 | 2.72 [1.83-4.05] | <0.0001 | 1.10 [0.73-1.65] | 0.8 |  |
| Italy | 1032 | 1.59 [1.08-2.34] | 0.1 | 1.14 [0.77-1.69] | 0.7 | 2.50 [1.70-3.67] | <0.0001 | 1.03 [0.70-1.52] | 1.0 |  |
| Netherlands | 1032 | 1.19 [0.80-1.77] | 0.7 | 1.40 [0.94-2.08] | 0.4 | 4.26 [2.88-6.31] | <0.0001 | 1.21 [0.81-1.79] | 0.7 |  |
| Poland | 1160 | 1.26 [0.87-1.82] | 0.5 | 0.95 [0.65-1.37] | 0.9 | 2.03 [1.41-2.92] | 0.002 | 1.61 [1.12-2.32] | 0.08 |  |
| Portugal | 1059 | 1.86 [1.27-2.74] | 0.02 | 2.32 [1.58-3.40] | 0.0003 | 6.99 [4.75-10.27] | <0.0001 | 1.68 [1.15-2.47] | 0.07 |  |
| Spain | 1000 | 0.81 [0.55-1.20] | 0.6 | 1.77 [1.20-2.61] | 0.04 | 3.00 [2.04-4.41] | <0.0001 | 1.17 [0.79-1.72] | 0.7 |  |
| Switzerland | 1088 | 1.08 [0.74-1.56] | 0.9 | 1.94 [1.34-2.80] | 0.006 | 4.23 [2.93-6.10] | <0.0001 | 0.99 [0.68-1.44] | 1.0 |  |
| United Kingdom | 1000 | 1.32 [0.89-1.95] | 0.5 | 1.97 [1.34-2.89] | 0.008 | 4.21 [2.86-6.20] | <0.0001 | 1.25 [0.85-1.85] | 0.6 |  |
| **Pizzas** | | | | | | | | | |  |
| All^c^ | 11896 | 1.16 [0.99-1.36] | 0.2 | 1.34 [1.14-1.57] | 0.004 | 2.22 [1.89-2.59] | <0.0001 | 1.05 [0.89-1.23] | 0.8 | |
| Belgium | 952 | 0.98 [0.58-1.64] | 1.0 | 1.84 [1.11-3.06] | 0.2 | 2.43 [1.47-4.01] | 0.02 | 0.81 [0.48-1.37] | 0.8 | |
| Bulgaria | 960 | 1.77 [1.08-2.88] | 0.2 | 0.95 [0.59-1.54] | 1.0 | 2.34 [1.43-3.82] | 0.02 | 1.35 [0.83-2.19] | 0.6 | |
| Denmark | 905 | 0.83 [0.50-1.37] | 0.8 | 1.29 [0.79-2.13] | 0.7 | 1.31 [0.80-2.14] | 0.6 | 0.78 [0.47-1.29] | 0.7 | |
| France | 979 | 1.77 [1.08-2.91] | 0.2 | 1.97 [1.20-3.25] | 0.1 | 2.71 [1.65-4.43] | 0.009 | 1.82 [1.11-3.01] | 0.2 | |
| Germany | 979 | 0.96 [0.57-1.59] | 1.0 | 1.45 [0.88-2.37] | 0.5 | 1.84 [1.13-3.01] | 2 | 0.94 [0.56-1.57] | 1.0 | |
| Italy | 1022 | 1.32 [0.80-2.17] | 0.6 | 1.13 [0.68-1.86] | 0.9 | 2.10 [1.29-3.41] | 0.06 | 0.90 [0.54-1.49] | 0.9 | |
| Netherlands | 972 | 1.57 [0.94-2.62] | 0.4 | 1.44 [0.87-2.40] | 0.5 | 2.66 [1.62-4.38] | 0.01 | 1.05 [0.63-1.74] | 1.0 | |
| Poland | 1128 | 0.89 [0.57-1.40] | 0.9 | 0.69 [0.44-1.09] | 0.4 | 1.61 [1.04-2.50] | 0.2 | 1.24 [0.79-1.93] | 0.7 | |
| Portugal | 1031 | 1.36 [0.84-2.19] | 0.6 | 1.43 [0.89-2.30] | 0.5 | 4.28 [2.71-6.76] | <0.0001 | 1.22 [0.75-1.96] | 0.8 | |
| Spain | 994 | 0.73 [0.43-1.22] | 0.6 | 1.68 [1.03-2.73] | 0.2 | 2.11 [1.30-3.42] | 0.06 | 0.72 [0.43-1.20] | 0.6 | |
| Switzerland | 1034 | 1.12 [0.69-1.84] | 0.9 | 1.28 [0.78-2.08] | 0.7 | 2.26 [1.41-3.61] | 0.02 | 0.93 [0.56-1.54] | 1.0 | |
| United Kingdom | 940 | 1.21 [0.72-2.04] | 0.8 | 1.87 [1.12-3.10] | 0.2 | 2.41 [1.46-3.98] | 0.02 | 1.05 [0.63-1.76] | 1.0 | |
| **Cakes** | | | | | | | | | |  |
| All^c^ | 12142 | 1.72 [1.42-2.09] | <0.0001 | 2.54 [2.09-3.07] | <0.0001 | 4.82 [3.98-5.82] | <0.0001 | 1.82 [1.50-2.21] | <0.0001 | |
| Belgium | 991 | 1.61 [0.92-2.82] | 0.3 | 3.24 [1.89-5.56] | 0.0003 | 6.35 [3.75-10.77] | <0.0001 | 1.69 [0.96-2.96] | 0.3 | |
| Bulgaria | 1003 | 2.94 [1.68-5.15] | 0.002 | 1.51 [0.87-2.63] | 0.4 | 2.93 [1.67-5.13] | 0.002 | 1.63 [0.94-2.84] | 0.3 | |
| Denmark | 964 | 1.68 [1.04-2.71] | 0.2 | 2.82 [1.75-4.56] | 0.0003 | 4.26 [2.66-6.82] | <0.0001 | 1.62 [1.00-2.61] | 0.2 | |
| France | 996 | 2.04 [1.19-3.49] | 0.07 | 4.90 [2.92-8.22] | <0.0001 | 9.03 [5.4-15.11] | <0.0001 | 2.18 [1.27-3.74] | 0.04 | |
| Germany | 976 | 2.01 [1.14-3.53] | 0.1 | 4.12 [2.38-7.15] | <0.0001 | 5.37 [3.11-9.28] | <0.0001 | 2.18 [1.23-3.87] | 0.06 | |
| Italy | 1028 | 1.59 [0.99-2.55] | 0.2 | 1.29 [0.80-2.07] | 0.7 | 2.31 [1.45-3.68] | 0.005 | 1.25 [0.78-2.01] | 0.7 | |
| Netherlands | 1019 | 1.31 [0.78-2.19] | 0.7 | 2.00 [1.21-3.32] | 0.06 | 5.46 [3.35-8.90] | <0.0001 | 1.94 [1.17-3.21] | 0.07 | |
| Poland | 1133 | 1.85 [1.16-2.97] | 0.07 | 1.31 [0.81-2.12] | 0.7 | 2.83 [1.78-4.51] | 0.0002 | 2.38 [1.49-3.81] | 0.003 | |
| Portugal | 1038 | 2.26 [1.41-3.61] | 0.007 | 3.34 [2.10-5.32] | <0.0001 | 7.65 [4.81-12.16] | <0.0001 | 2.31 [1.44-3.69] | 0.005 | |
| Spain | 975 | 1.14 [0.68-1.91] | 0.9 | 3.09 [1.87-5.11] | 0.0002 | 4.72 [2.88-7.75] | <0.0001 | 2.10 [1.26-3.48] | 0.04 | |
| Switzerland | 1039 | 1.33 [0.82-2.17] | 0.6 | 3.11 [1.96-4.95] | <0.0001 | 6.96 [4.40-11.00] | <0.0001 | 1.47 [0.91-2.38] | 0.4 | |
| United Kingdom | 980 | 1.87 [1.10-3.17] | 0.1 | 2.64 [1.57-4.44] | 0.003 | 7.53 [4.55-12.46] | <0.0001 | 2.18 [1.29-3.68] | 0.04 | |
| **Breakfast cereals** | | | | | | | | | |  |
| All^c^ | 11595 | 1.14 [0.98-1.33] | 0.2 | 1.31 [1.13-1.52] | 0.003 | 2.36 [2.05-2.73] | <0.0001 | 0.96 [0.83-1.12] | 0.6 | |
| Belgium | 917 | 1.07 [0.62-1.86] | 1.0 | 1.49 [0.87-2.54] | 0.7 | 1.87 [1.09-3.19] | 0.3 | 0.67 [0.38-1.18] | 0.8 | |
| Bulgaria | 964 | 1.50 [0.80-2.80] | 0.8 | 1.35 [0.72-2.53] | 0.8 | 2.02 [1.09-3.75] | 0.3 | 0.94 [0.50-1.78] | 1.0 | |
| Denmark | 978 | 0.90 [0.56-1.45] | 1.0 | 1.11 [0.70-1.77] | 1.0 | 1.66 [1.05-2.62] | 0.4 | 0.76 [0.47-1.23] | 0.8 | |
| France | 892 | 0.88 [0.53-1.46] | 1.0 | 1.19 [0.72-1.95] | 0.9 | 1.93 [1.19-3.15] | 0.1 | 0.89 [0.54-1.47] | 1.0 | |
| Germany | 879 | 1.45 [0.83-2.52] | 0.8 | 1.68 [0.98-2.90] | 0.4 | 2.55 [1.49-4.34] | 0.02 | 0.95 [0.54-1.68] | 1.0 | |
| Italy | 963 | 1.53 [0.88-2.65] | 0.7 | 1.11 [0.64-1.93] | 1.0 | 2.72 [1.61-4.60] | 0.008 | 0.88 [0.50-1.53] | 1.0 | |
| Netherlands | 931 | 0.97 [0.57-1.66] | 1.0 | 1.03 [0.60-1.76] | 1.0 | 3.48 [2.11-5.73] | 0.0001 | 0.94 [0.55-1.61] | 1.0 | |
| Poland | 1115 | 1.62 [1.01-2.60] | 0.4 | 1.25 [0.78-2.01] | 0.8 | 1.90 [1.19-3.05] | 0.1 | 1.52 [0.95-2.45] | 0.5 | |
| Portugal | 1027 | 1.74 [1.04-2.92] | 0.4 | 2.07 [1.25-3.44] | 0.1 | 4.76 [2.92-7.77] | <0.0001 | 1.61 [0.96-2.70] | 0.5 | |
| Spain | 940 | 0.64 [0.39-1.06] | 0.5 | 0.95 [0.58-1.55] | 1.0 | 1.98 [1.25-3.14] | 0.09 | 0.91 [0.56-1.48] | 1.0 | |
| Switzerland | 1006 | 0.88 [0.55-1.41] | 1.0 | 1.41 [0.90-2.22] | 0.7 | 2.63 [1.69-4.07] | 0.001 | 0.79 [0.49-1.26] | 0.8 | |
| United Kingdom | 983 | 1.30 [0.81-2.10] | 0.8 | 1.62 [1.02-2.58] | 0.4 | 2.74 [1.74-4.33] | 0.001 | 0.92 [0.57-1.50] | 1.0 | |

^a^ The reference of the multivariate ordinal logistic regression for the categorical variable ‘FoPL’ was the Reference Intakes label. The multivariate model was adjusted on sex, age, educational level, level of income, responsibility for grocery shopping, self-estimated diet quality, and self-estimated nutrition knowledge level.

^b^ P-values were obtained after a False Discovery Rate correction to consider multiple testing.

^c^ The model on the overall sample was conducted using a random effect on the FoPL variable.

HSR: Health Star Rating system; MTL: Multiple Traffic Lights; OR: Odds Ratio; CI: Confidence Interval.

**Table S9. Associations^a^ between FoPLs and the change in participants’ ability to correctly rank the nutritional quality of foods, adjusted on food category purchasing frequency**

| **Food categories** | **N** | **HSR** | | **MTL** | | **Nutri-Score** | | **Warning symbol** | |
| --- | --- | --- | --- | --- | --- | --- | --- | --- | --- |
|  |  | **OR (95% CI)** | **P^b^** | **OR (95% CI)** | **P^b^** | **OR (95% CI)** | **P^b^** | **OR (95% CI)** | **P^b^** |
| **All countries^c^** | | | | | | | | | |
| Pizza | 11896 | 1.19 [1.03-1.39] | 0.1 | 1.33 [1.14-1.54] | 0.003 | 2.06 [1.77-2.39] | <0.0001 | 1.08 [0.93-1.26] | 0.6 |
| Cakes | 12142 | 1.62 [1.34-1.96] | <0.0001 | 2.26 [1.87-2.73] | <0.0001 | 4.16 [3.45-5.02] | <0.0001 | 1.72 [1.43-2.08] | <0.0001 |
| Breakfast cereals | 11595 | 1.17 [1.02-1.34] | 0.06 | 1.29 [1.13-1.48] | 0.002 | 2.18 [1.91-2.50] | <0.0001 | 1.01 [0.88-1.16] | 1.0 |

^a^ The reference of the multivariate ordinal logistic regression for the categorical variable ‘FoPL’ was the Reference Intakes label. The multivariate model was adjusted on sex, age, educational level, level of income, responsibility for grocery shopping, self-estimated diet quality, self-estimated nutrition knowledge level, and the food category purchasing frequency.

^b^ P-values were obtained after a False Discovery Rate correction to consider multiple testing.

^c^ The model on the overall sample was conducted using a random effect on the FoPL variable.

HSR: Health Star Rating system; MTL: Multiple Traffic Lights; OR: Odds Ratio; CI: Confidence Interval.
